# Evaluation of Wound-Healing Potential of Plant Extracts via Picture Fuzzy Multi-Attribute Decision-Making

**DOI:** 10.1021/acsomega.6c08040

**Published:** 2026-08-17

**Authors:** Firdevs Mert Sivri, Sait Gül

**Affiliations:** † Faculty of Pharmacy, Department of Basic Pharmaceutical Sciences, 52994 Suleyman Demirel University, 32200 Isparta, Turkey; ‡ Faculty of Engineering and Natural Sciences, Department of Industrial Engineering, 52946 Bahçeşehir University, 34353 Istanbul, Turkey

## Abstract

The rising number of chronic wounds across the world makes the creation of efficient, environmentally friendly and cost-effective wound healing biopolymers an urgent need in the sphere of biopharmaceuticals. However, the selection of the most appropriate candidate from many natural plant extracts with complex and heterogeneous phytochemical composition is a difficult task of multiattribute decision-making (MADM) because of uncertainties, hesitations in expert judgments, and incomplete experimental data. A new hybrid approach of MADM with the use of picture fuzzy sets (PFS) is suggested in this paper as a realistic model of human logical judgment with membership degrees that cover positive, negative, neutral, and refusal aspects. Within the context of this study, the PiF-DEMATEL method is used to estimate causal relationships between eight attributes related to tissue repairing performance and attribute weights, while the PiF-PIV method that has been elaborated for the first time in the literature in this study is proposed as a tool for ranking ten different plant extracts. According to the attribute analysis, the “anti-inflammatory effect” (0.150) and “cell regeneration” (0.147) attributes, belonging to the group of physiological response attributes, were shown to have the highest importance, whereas the “Phytochemical content” (0.145) criterion was revealed to be the most dominating causative factor controlling the underlying biochemical engine. *Centella asiatica* (*C. asiatica*) turned out to be the most stable and efficient wound-healing candidate, being immediately followed by *Hypericum perforatum* (*H. perforatum*) and *Calendula officinalis* (*C. officinalis*). Sensitivity analysis including alternative weighting and ranking approaches showed the minimum value of rank correlation coefficient at 96.4%, which proves high reliability, mathematical validity, and robustness of the PiF-DEMATEL-PIV hybrid model. Thus, this study proposes a potent and economic guide for engineering that helps to avoid losses caused by trial-and-error during preliminary laboratory stages of creation of advance *H. perforatum* d wound care technologies, e.g., electrospun nanofibers and polysaccharide hydrogels.

## Graphical abstract

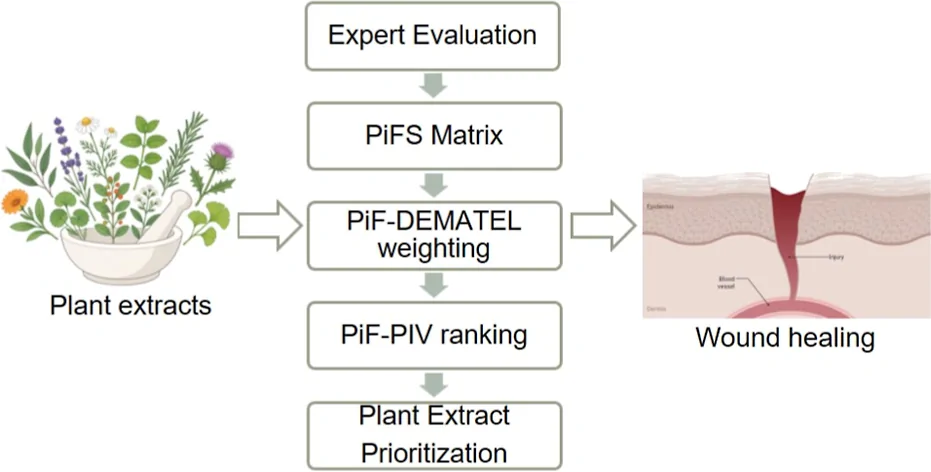

## 1 Introduction

Wound healing is a carefully coordinated biological process in which cells move in, multiply, and rebuild damaged tissue through four overlapping phases: hemostasis, inflammation, proliferation, and maturation.^1–3^ Most minor injuries heal on their own, but large wounds and chronic lesions are much harder to repair and remain a major global health problem.^4–7^ Aging, poor blood supply (ischemia), and systemic diseases like diabetes often slow or derail repair, creating “silent epidemics” that affect many people worldwide and place heavy economic and psychological strain on healthcare systems.^8–10^ In conditions such as diabetic foot ulcers or severe burns, prolonged hyper-inflammation often paired with multidrug-resistant infections can lead to complications that conventional treatments struggle to resolve efficiently.^1,11–13^

To address these challenges, modern biopharmaceutical research is increasingly revisiting traditional medical knowledge, where medicinal plants have long played an important role in supporting tissue repair.^2,11,12^ Plant-derived bioactive compounds such as phenolics, flavonoids, alkaloids, saponins, tannins, and terpenoids have shown strong potential to shape the wound environment in beneficial ways.^2,12^ They may help by neutralizing reactive oxygen species (ROS), reducing pro-inflammatory cytokines, and boosting healing signals such as VEGF and TGF-β that support angiogenesis and collagen deposition.^1,2,12^

Many plant extracts have been studied for wound-healing activity, ranging from the re-epithelialization benefits of *Aloe vera* and *Centella asiatica* to the antimicrobial and anti-inflammatory actions reported for *Curcuma longa*, *Punica granatum*, and *Aronia melanocarpa*.^1,2,9^ Still, identifying the “best” extract for advanced medical use is not simple because plants vary in composition, solubility, and batch-to-batch consistency.^1,2,9^ The challenge grows when extracts are integrated into engineered delivery platforms—such as electrospun nanofibers or polysaccharide hydrogels—where researchers must balance biological performance with design constraints like surface area, mechanical stability, and durability.^1,4,7,13^

To navigate this multifactor decision space, researchers use multi-attribute decision-making (MADM) methods.^3,6,7^ Techniques such as the analytic hierarchy process (AHP) and TOPSIS offer structured, quantitative ways to rank options across many competing criteria.^6,7^ More recent MADM approaches aim to represent expert judgment more realistically by capturing uncertainty, hesitancy, and partial agreement.^5–7^ This makes it possible to compare plant extracts more rigorously even when expert input and experimental evidence are incomplete or variable.^6,7^

Although numerous experimental studies exist in the literature regarding the wound healing potential of plant extracts, a detailed study that incorporates decision-making methods, particularly expert opinions, has not been found in this area. This study aims to systematically compare the wound-healing potential of multiple plant extracts using hybrid MADM methodologies.^6,11^ By combining evidence-based data with advanced decision-analysis tools, the work seeks to build a more objective framework for selecting herbal candidates for clinical application.^6,11^ Ultimately, the goal is to support the development of next-generation wound care technologies that are effective while also being economically and environmentally sustainable.^7,9,11^

MADM supports engineering and managerial decision-making by providing quantitative tools for selecting the best option among alternatives evaluated against multiple attributes. Because many problems in engineering, business, finance, energy, textile, and related fields involve diverse and changing criteria, they can be effectively modeled as MADM problems. In this context, MADM deserves attention as a useful analytical approach for assessing plant extracts for their wound-healing potential.

MADM-based decision analysis typically follows three consecutive stages. The process begins by developing a decision model that defines the relevant attributes, alternatives, experts, constraints, and data types. In the second stage, the collected information is organized into a decision matrix using data obtained from appropriate sources. Finally, this decision matrix is evaluated through suitable MADM methods. During data collection, experts are often consulted to form a decision matrix that includes nonquantitative performance ratings. When sufficient objective data obtained through experimental measurement is unavailable, linguistic subjective assessments are accepted as representations of experts’ preferences, judgments, and knowledge. In this context, fuzzy set concepts serve as effective supporting tools.

Zadeh^14^ established the concept of ordinary fuzzy sets, which can be used to represent human judgments. In these sets, the membership degree (μ) ranges from 0 to 1 and indicates the extent to which a judgment is supported or agreed with. In this way, it mainly reflects a positive aspect of evaluation. Over the past six decades, several fuzzy set extensions have been proposed to better address uncertainty and ambiguity. Atanassov^15^ introduced intuitionistic fuzzy sets (IFS), adding the nonmembership degree (ν) to express the level of disagreement in a judgment. He also defined another component, hesitancy or indeterminacy, which represents the neutral or uncertain part of expert opinion and is calculated as π = 1 – μ – ν. Therefore, IFS became the first three-dimensional fuzzy set model, capable of capturing membership, nonmembership, and hesitancy in expert evaluations. After the introduction of IFS, several fuzzy set extensions were proposed, including Pythagorean fuzzy sets (PFS),^16^ q-rung orthopair fuzzy sets (q-ROFS),^17^ Fermatean fuzzy sets (FFS),^18^ spherical fuzzy sets (SFS),^19^ and picture fuzzy sets (PiFS).^20^

In this work, the concept of PiFS is adopted due to its comprehensive structure, which incorporates membership, nonmembership, hesitancy, and refusal degrees. PiFS, introduced by Cuong and Kreinovich,^20^ is the only fuzzy set framework that accounts for all four dimensions of human opinion. Specifically, membership and nonmembership degrees represent agreement and disagreement, respectively, while hesitancy reflects uncertainty or indecision, and refusal indicates an unwillingness to express an opinion. This structure provides a more realistic representation of real-world decision-making situations, particularly when information is incomplete, uncertain, or imprecise.^21^

Among many others, this study prefers to use a DEMATEL approach for weighting attributes and a PIV method to assess and rank the alternatives of plant extracts. Instead of crisp (traditional) versions of these methods, PiF extensions are utilized. PiF concept models human opinion with four dimensions and is capable of handling any natural uncertainty or ambiguity. The subjective perspective of this study can be supported by this kind of powerful representation system of human logic.

Following a comprehensive literature review and consultations with a group of five experts, the key attributes that may significantly influence the performance evaluation process were obtained. Subsequently, the available plant extracts were identified, and the required data were obtained from the same five experts. The linguistic term set, which is designed for PiF environments, is not utilized here because of its limiting effect on the representation of the judgment in its full potential. Instead, the data collection process developed by Gül^22^ is utilized. This unique data gathering framework was inspired by Joshi^23^ and Joshi,^24^ and its applicability was demonstrated by Gül and Sivri^7^ in ideal natural waste sorting problem for sustainable production of nanofibers via electrospinning. Besides introducing this unique data gathering framework’s practicality under PiF conditions, Gül and Sivri^7^ also presented how fuzzy MADM approaches may provide performance evaluation and ranking analytics in sustainable production.

PiF-DEMATEL method was selected for subjective attribute weighting since considering potential interrelationships among attributes is essential in this problem. DEMATEL is particularly useful because of its ability to detect causal relationships among attributes and derive the corresponding weights by processing these relationships. The PiF-DEMATEL-based attribute weights and the performance evaluations obtained from the judgments of the experts regarding the performance evaluations of plant extracts with respect to attributes were then analyzed together using the PiF-PIV method.

Since only a limited number of fuzzy extensions of PIV currently exist and there has been no PiF extension of the Proximity Indexed Value (PIV) method yet in the literature, a PiF-PIV method was developed. This theoretical contribution aims to benefit from the superior ability of the PiF concept in modeling human judgments in a more comprehensive manner.^25^ The literature on PIV method has been evolving every day, mainly due to its practicality, straightforward implementation, robustness against the rank reversal problem, and applicability for researchers who may not have extensive experience in MADM methods. The validity and robustness of the proposed PiF-extended DEMATEL-PIV hybrid MADM model were demonstrated by comparing the resulting rankings of plant extracts with those generated by different weighting (entropy-based weighting and CRITIC) and ranking (ARAS and TOPSIS) scenarios.

The main contributions of this study to literature may be summarized as follows:

i. This study aims to develop a more comprehensive subjective decision-making model for prioritizing the attributes according to their effects’ significance levels in assessing the plant extracts’ wound-healing potentials. Instead of costly physical prearrangements, such a model can provide a good and cheaper starting point before organizing well-established experiments.
ii. PiF-DEMATEL enables both the interactions among attributes and their weights to be determined based on expert judgments. Although PiF-DEMATEL was previously introduced by Gül^22^ and Gül and Sivri,^7^ this study presents another application in sustainable production of recycled components.
iii. The PIV method, as a relatively new one in the MADM field, is extended into the PiF conditions for the first time in literature. With its unique data gathering process, it has potential to be used

The paper is organized as follows: Section 2 provides the technical characteristics of PiFS concept and mathematics, a data collection system that is unique for PiF, and the PiF extension of DEMATEL; Section 3 presents the newly proposed algorithm of PiF extension of PIV; Section 4 explores the definition of the performance evaluation problem of plant extracts with respect to their wound-healing capabilities, and also calculation details of PiF-DEMATEL-PIV are presented with validity checking results; Section 5 discusses the ranking results; and Section 6 concludes the paper with the general findings, limitations, and future research propositions.

## 2 Technical Preliminaries

### 2.1 Picture Fuzzy Sets

Zadeh’s^14^ fuzzy set (FS) theory represents one of the earliest efforts in the literature to help decision analysts address linguistic vagueness and uncertainty in expert judgments during decision-making processes. FSs express an expert’s assessment using only a membership degree. Atanassov^15^ later expanded this concept by introducing intuitionistic fuzzy sets (IFS), which incorporate an additional parameter, the nonmembership degree, to reflect an expert’s disagreement. Since the emergence of FSs and IFSs, many advanced extensions have been developed. Pythagorean fuzzy sets,^16^ q-rung orthopair fuzzy sets,^17^ Fermatean fuzzy sets,^18^ and linear Diophantine fuzzy sets^26^ have enhanced the representation of human evaluations by enabling separate definitions of positive and negative membership degrees. Nevertheless, these approaches do not explicitly include a hesitancy component. In contrast, neutrosophic sets,^27^ spherical fuzzy sets,^19^ and picture fuzzy sets (PiFS) introduced hesitation as an independent degree.^20^

The PiFS concept can be regarded as a broader and more comprehensive extension of fuzzy sets, designed to represent uncertain and vague expert evaluations in MADM problems. PiFSs are characterized by three independently assignable degrees: positive membership degree (α), neutral membership degree (η), and negative membership degree (β), each ranging between 0 and 1. The positive and negative membership degrees, α and β, correspond to the meanings used in FSs and IFSs. The neutral membership degree (η) reflects the expert’s neutral stance toward a given proposition and represents the degree of hesitation. PiFSs are subject to the condition that the sum α + η + β lies within the unit interval [0,1]. Since this sum may be less than 1, the remaining value, expressed as 1 – (α + η + β), is defined as the refusal degree and denoted by π. This component indicates the expert’s decision to withhold or refuse to provide an opinion or judgment. Thus, a PiFS is a generalized fuzzy structure composed of four elements, three of which can be independently assigned by the expert, and it satisfies two main conditions: (i) α,η,β, and π are all within [0,1], and (ii) α + β + η + π = 1.

Cuong^28^ explains the concept of PiFSs using a voting example. In this setting, voters can be classified into four categories according to their behavior: those who vote in favor of a candidate, those who remain neutral or abstain, those who vote against the candidate, and those who refuse to participate in the voting process. Garg^29^ presents another illustration in which a decision analyst consults an expert to determine the degrees of truth, falsity, and neutrality associated with a particular statement. In PiFS notation, this evaluation can be represented as (α,η,β) = (0.3, 0.2, 0.4). Since the total of these values is 0.9, the remaining portion is 0.1, which corresponds to the refusal degree and is denoted by π. This example highlights that conventional FSs and IFSs are not sufficiently capable of modeling such a situation.

Definition 1.^28^ A PiFS *P* on a universe of *X* is defined as follows

1 P={(x,α(x),η(x),β(x))|x∈X}

where α­(*x*) denotes positive, η­(*x*) is neutral, and β­(*x*) denotes negative membership degrees satisfying the conditions: 0 ≤ α­(*x*),η­(*x*),β­(*x*) ≤ 1 and 0 ≤ α­(*x*) + η­(*x*) + β­(*x*)≤1. π­(*x*) = 1 – α­(*x*) – η­(*x*) – β­(*x*) is the refusal membership degree of *x* on *X*. As defined, when π­(*x*) = 0, *P* becomes IFS and when π­(*x*) = η­(*x*) = 0, *P* is equalized to FS.

Definition 2.^7^ A picture fuzzy number (PiFN) is a collection of *P* with the following conditions:

i. A PiFN is defined shortly by (α,η,β),
ii. 0 ≤ α,η,β ≤ 1
iii. 0 ≤ α + η + β ≤ 1
iv. π = 1 – α – η – β is the refusal degree.

Definition 3.^28,30^ Let Γ = (α_Γ_,η_Γ_,β_Γ_) and ψ = (α_ψ_,η_ψ_,β_ψ_) be two PiFNs, then.

1. Γ∨ψ = ⟨max­(α_Γ_,α_ψ_),min­(η_Γ_,η_ψ_),min­(β_Γ_,β_ψ_)⟩
2. Γ∧ψ = ⟨min­(α_Γ_,α_ψ_),min­(η_Γ_,η_ψ_),max­(β_Γ_,β_ψ_)⟩
3. Γ^c^ = ⟨β_Γ_,η_Γ_,α_Γ_⟩.
4. Γ⊕ψ = (1–(1 – α_Γ_)­(1 – α_ψ_),η_Γ_η_ψ_,(η_Γ_ + β_Γ_)­(η_ψ_ + β_ψ_) – η_Γ_η_ψ_)
5. Γ⊗ψ = ((α_Γ_ + η_Γ_)­(α_ψ_ + η_ψ_) – η_ψ_η_ψ_),(η_Γ_η_ψ_,1–(1 – β_Γ_)­(1 – β_ψ_))
6.
$\omega \Gamma =\left(1-{\left(1-\alpha_{\Gamma }\right)}^{\omega },{\left(\eta_{\Gamma }\right)}^{\omega },{\left(\eta_{\Gamma }+\beta_{\Gamma }\right)}^{\omega }-{\left(\eta_{\Gamma }\right)}^{\omega }\right)$

7.
$\Gamma^{\omega }=\left({\left(\alpha_{\Gamma }+\eta_{\Gamma }\right)}^{\omega }-{\left(\eta_{\Gamma }\right)}^{\omega },{\left(\eta_{\Gamma }\right)}^{\omega },1-{\left(1-\beta_{\Gamma }\right)}^{\omega }\right)$

Definition 4. Let Γ = (α_Γ_,η_Γ_,β_Γ_) be a PiFN. A score function (sc­(Γ)) is defined by Xu et al.^31^ as follows

2 $$sc\left(\Gamma \right)=\alpha_{\Gamma }+\frac{\eta_{\Gamma }}{2}+\frac{1-\alpha_{\Gamma }-\eta_{\Gamma }-\beta_{\Gamma }}{2}\left(1+\alpha_{\Gamma }-\beta_{\Gamma }\right)$$

Definition 5.^20^ Let Γ = (α_Γ_,η_Γ_,β_Γ_) and ψ = (α_ψ_,η_ψ_,β_ψ_) be two PiFNs, then Euclidean and Hamming distance measures are defined as follows

3 $$d_{E}\left(\Gamma ,\psi \right)={\left[{\left(\alpha_{\Gamma }-\alpha_{\psi }\right)}^{2}+{\left(\eta_{\Gamma }-\eta_{\psi }\right)}^{2}+{\left(\beta_{\Gamma }-\beta_{\psi }\right)}^{2}\right]}^{1/2}$$

4 $$d_{H}\left(\Gamma ,\psi \right)=\left|\alpha_{\Gamma }-\alpha_{\psi }\right|+\left|\eta_{\Gamma }-\eta_{\psi }\right|+\left|\beta_{\Gamma }-\beta_{\psi }\right|$$

Definition 6.^32^ For any Γ­(*x*_*i*_)∈PiFS­(*X*), Εn/PiFS­(*X*) → [0,1] is the entropy measure.

5 $$Εn=\frac{1}{n}\sum_{i=1}^{n}\frac{1-\left|\alpha_{\Gamma }\left(x_{i}\right)-\beta_{\Gamma }\left(x_{i}\right)\right|-\left|\eta_{\Gamma }\left(x_{i}\right)-\pi_{\Gamma }\left(x_{i}\right)\right|}{1+\left|\alpha_{\Gamma }\left(x_{i}\right)-\beta_{\Gamma }\left(x_{i}\right)\right|+\left|\eta_{\Gamma }\left(x_{i}\right)-\pi_{\Gamma }\left(x_{i}\right)\right|},\forall x_{i}\in X$$

### 2.2 Data Collection and Aggregation System Designed for PiFNs

The basics of this unique data collection system for PiFN were introduced by Joshi.^23,24^ An extended version was developed by Gül^32^ for PiFN-based DEMATEL with the aim of obtaining the attribute weights, and its applicability was demonstrated through a case study on education quality evaluation. Gül and Sivri^7^ improved it for alternative evaluations and presented its practicality in sorting waste materials for sustainable production of nanofibers via electrospinning.

Suppose that expert *e* expresses his/her evaluations regarding the performance of each alternative *i* with respect to each attribute *j*, where (*e* = 1,···, *E*; *i* = 1,···,*m; j* = 1,···,*n*), using the linguistic scale described below. Although the direct use of a predefined set of linguistic terms is one possible approach, such a representation may be inadequate because it can restrict experts’ freedom of choice. Moreover, experts may find this type of scale difficult to interpret.

As a general framework, the components of PiFN may be interpreted easily as a voting system. Voting in favor of a candidate with a positive attitude corresponds to α. Conversely, not wishing for a candidate to be elected reflects a negative stance, which is represented by β. Being undecided about whether to vote for or against a candidate, or maintaining a neutral view toward that candidate, can be expressed through η. Finally, distrust in the voting system and/or refusing to vote for the candidate for any reason corresponds to π.

As these four elements represent the possible characteristics of an expert evaluation, the linguistic scale presented to the experts consists of only four terms: YES, NO, ABSTAIN, and REFUSE. In addition, experts are allowed to assign a degree to the linguistic term YES so that they can express the intensity of their agreement-based opinions. The details of this data collection procedure can be illustrated through the following examples:

- If an expert believes that alternative *i* achieves the highest possible performance level with respect to attribute *j*, this assessment is expressed as YES with a degree of 1.00, i.e., YES (1.00).
- If an expert considers that alternative *i* partially satisfies the expectations for attribute *j*, for instance at a level of 60%, this degree of agreement is represented as YES (0.60).
- If the performance of alternative *i* makes no contribution to the satisfaction of attribute *j*, the expert’s negative opinion is represented by a linguistic term NO, indicating complete disagreement.
- If an expert is uncertain about the performance of alternative *i* regarding attribute *j* and is unwilling to provide either a positive or negative judgment, the term ABSTAIN is used to reflect this indeterminate evaluation.
- If an expert has no knowledge about the performance of alternative *i* with respect to attribute *j*, they may choose not to express any opinion. In this case, the linguistic term REFUSE is used to represent this situation.

Experts’ linguistic assessments of the performance of alternative *i* with respect to attribute (*j*), expressed as YES, NO, ABSTAIN, or REFUSE, are first collected as explained above. These assessments are then aggregated to construct the expert evaluation matrix used in MADM methods. A statistical aggregation procedure for combining expert opinions was initially proposed by Joshi,^23,24^ following a voting-based logic. This approach was also adopted in a similar manner by Arya and Kumar^33^ and Jovčić et al.^34^ Later, Gül^22^ incorporated a gradation mechanism into positive responses, namely the membership degree, and demonstrated the practicality of the modified system in an educational quality evaluation problem using PiF-DEMATEL. However, since DEMATEL is a pairwise comparison-based subjective attribute weighting approach, the first version proposed by Gül^22^ was modified for alternative ranking and sorting by Gül and Sivri.^7^

The aggregated expert evaluation matrix is presented by $\mathrm{\Gamma ̃}={\left[{\mathrm{\gamma ̃}}_{ij}\right]}_{m*n}=\left[(\alpha_{ij},\eta_{ij},\beta_{ij})\right]$ where *i* = 1,···,*m* ; *j* = 1,···,*n*. (α_*ij*_,η_*ij*_,β_*ij*_) depicts the positive, neutral, and negative membership degrees represented by YES (agreement level), ABSTAIN, and NO, respectively. The refusal degree symbolized by the REFUSE linguistic term should hold π_*ij*_ = 1 – α_*ij*_ – η_*ij*_ – β_*ij*_.

The components of $\mathrm{\Gamma ̃}={\left[{\mathrm{\gamma ̃}}_{ij}\right]}_{m*n}$ are computed by utilizing eqs 6–9), respectively.

6 $$\alpha_{ij}=\frac{\sum_{e=1}^{E}{YES}_{ij}\left(\mu_{ij}^{e}\right)}{E}$$

7 $$\eta_{ij}=\frac{\delta_{{ABSTAIN}_{ij}}}{E}+\frac{\frac{\delta_{{YES}_{ij}}}{E}-\alpha_{ij}}{2}$$

8 $$\beta_{ij}=\frac{\delta_{{NO}_{ij}}}{E}$$

9 $$\pi_{ij}=\frac{\delta_{{REFUSE}_{ij}}}{E}+\frac{\frac{\delta_{{YES}_{ij}}}{E}-\alpha_{ij}}{2}$$

where δ shows the number of votes; YES_*ij*_(μ_*ij*_^e^) shows the preference grade within the positive idea (YES) of the expert *e*.

There are two components in each of eqs 10 and 12. The first component represents the average number of ABSTAIN or REFUSE votes, while the second component corresponds to one-half of the unassigned degree remaining from the YES response (YES_*ij*_(μ_*ij*_^e^)). Initially, the average of the experts’ positive evaluations, including both complete and partial opinions, is calculated. After this step, some preference degree may remain unassigned. Since the condition α_*ij*_ + η_*ij*_ + β_*ij*_ + π_*ij*_ = 1 must be satisfied for a PiFN, allocating this unassigned portion equally between the ABSTAIN and REFUSE degrees appears to be the most practical and reasonable approach. However, if the decision analyst can justify it, alternative distribution strategies may also be used. For instance, the entire unassigned part may be directly assigned to either the REFUSE or ABSTAIN degree. Illustrative numerical examples are provided in Section 4.

### 2.3 Picture Fuzzy Extension of DEMATEL (PiF-DEMATEL)

DEMATEL is a MADM method that relies on expert evaluations to identify the underlying causal relationships among attributes. In this study, we adopted PiF-DEMATEL for weighting attributes affecting the evaluation of plant extracts with respect to their wound-healing capability. The procedure consists of eight steps in total.^22^

#### 2.3.1 Step 1. Determining the Attributes and the Experts

Assume that there are *E* experts who can contribute their knowledge to solving a decision-making problem, and *n* attributes that may influence the final solution (*e* = 1,···,*E* ; *i*,*k* = 1,···,*n*; *i*≠*k*). Since the primary objective of DEMATEL is to identify the influences among these attributes, each expert *e* is requested to express their opinion about the possible influence between every pair of attributes (*i*,*k*). To do so, a linguistic evaluation scale is required to represent the experts’ judgments. In the PiF environment, these linguistic responses are expressed as YES, NO, ABSTAIN, and REFUSE, as they reflect the four possible forms of expert judgment that need to be captured. Therefore, a voting-based procedure is adopted in this approach. The details can be seen in Section 2.2.

#### 2.3.2 Step 2. Obtaining Direct PiF Influence Matrix

The influence of attribute *j* on attribute *k* is collected from the experts, resulting in *E* separate evaluation matrices, each with dimensions (n*n). These matrices must then be combined before starting the DEMATEL procedure. Section 2.2 provides us with the required calculations in eqs 6–9). These equations are designed for alternative evaluations so that the indices are updated as follows

10 $$\alpha_{jk}=\frac{\sum_{e=1}^{E}{YES}_{jk}\left(\mu_{jk}^{e}\right)}{E}$$

11 $$\eta_{jk}=\frac{\delta_{{ABSTAIN}_{jk}}}{E}+\frac{\frac{\delta_{{YES}_{jk}}}{E}-\alpha_{jk}}{2}$$

12 $$\beta_{jk}=\frac{\delta_{{NO}_{jk}}}{E}$$

13 $$\pi_{jk}=\frac{\delta_{{REFUSE}_{jk}}}{E}+\frac{\frac{\delta_{{YES}_{jk}}}{E}-\alpha_{jk}}{2}$$

According to these aggregation equations, the direct influence matrix $\mathrm{\Psi ̃}={\left[{\mathrm{\gamma ̃}}_{jk}\right]}_{n*n}=\left[(\alpha_{jk},\eta_{jk},\beta_{jk})\right]$ is revealed.

#### 2.3.3 Step 3. Obtaining Direct Influence Matrix

DEMATEL involves several matrix-based operations, including matrix inversion and matrix multiplication. However, when these operations are carried out independently, the PiF nature of the data is often distorted. To prevent such distortion, many researchers, such as Xie et al.,^35^ Abdel-Basset et al.,^36,37^ Al-Quran et al.,^38^ and Tan and Zhang,^39^ have adopted early defuzzification. Therefore, at this stage, defuzzifying the direct influence PiF evaluation matrix appears to be the most suitable approach for preserving the basic principles of the method.

The crisp influence of attribute *j* on attribute *k* is represented by **γ**_**jk**_, and the direct influence matrix is presented by Ψ = [**γ**_**jk**_]. In this matrix, each element of Ψ is defuzzified using eq 14, which originates from score function (eq 2).

14 $$\gamma_{jk}=\alpha_{jk}+\frac{\eta_{jk}}{2}+\frac{1-\alpha_{jk}-\eta_{jk}-\beta_{jk}}{2}\left(1+\alpha_{jk}-\beta_{jk}\right)$$

The remaining steps are identical to the traditional DEMATEL.^40,41^

#### 2.3.4 Step 4. Building Initial Direct Influence Matrix

$Ζ={\left[\varepsilon_{jk}\right]}_{n*n}$ is obtained by performing eq 15. *k* is the normalization index. Thus.

15 $$\varepsilon_{jk}=\frac{\gamma_{jk}}{k}where\;k=\max\left[\max_{j}\sum_{k=1}^{n}x_{jk},\max_{k}\sum_{j=1}^{n}x_{jk}\right]$$

#### 2.3.5 Step 5. Determining Total Influence Matrix

The total influence is the sum of direct influences (Ζ) and all the indirect influences (Ζ,^2^ Ζ,^3^,.., Ζ^∞^). A convergent solution is found as given in eq 16, exploiting the same understanding with absorbing Markov chains.

16 $$Τ={\left[\tau_{jk}\right]}_{n*n}=Ζ+{Ζ}^{2}+{Ζ}^{3}+...+{Ζ}^{\infty }=Ζ{\left(I-Ζ\right)}^{-1}$$

#### 2.3.6 Step 6. Computing Prominence and Relation Values

DEMATEL computes the row sum (*R*) and column sum (*C*) first.

17 $$R={\left[r_{j}\right]}_{n*1}=\left[\sum_{k=1}^{n}\tau_{jk}\right]$$

18 $$C={\left[c_{k}\right]}_{1*n}={\left[\sum_{j=1}^{n}\tau_{jk}\right]}^{\prime }$$

The row-sum vector (*R*) reflects the total influence exerted by attribute *j* on the other attributes and therefore represents its overall strength. In contrast, the column-sum vector (*C*) shows the extent to which attribute *k* is affected by the other attributes, indicating its overall weakness.

For (*j* = *k*) and *i,j* = 1,···,*n*, the value of (*R* + *C*), referred to as Prominence, expresses the total amount of influence both given and received by attribute *j*. Meanwhile, (*R*–*C*), known as Relation, indicates the net contribution of attribute *j* to the system. Based on the sign of the Relation value, attributes are categorized into either the cause group or the effect group.

a. If (*R*–*C* > 0), we can assume that attribute *j* generally affects other attributes and is therefore classified as a cause attribute.
b. If (*R*–*C* < 0), attribute *j* is generally affected by other attributes and is therefore classified as an effect attribute.

The attributes are interpreted in terms of their groups such that any improvement achieved in the cause group attributes potentially generates an indirect enhancement in the effect group attributes. Thus, when resources are limited, investment or any enhancement priority should be directed to the cause attributes.

#### 2.3.7 Step 7. Drawing Influential Relation Map (IRM)

The influence results obtained from DEMATEL are illustrated through an Influence Relation Map (IRM). In this map, the Prominence values are placed on the horizontal axis, while the Relation values are shown on the vertical axis. Attributes belonging to the cause group are positioned in the upper part of the IRM, whereas effect attributes are located in the lower part. Although the IRM can display the overall relationships among attributes, the number of visible connections increases as the complexity of the system grows. Therefore, to reduce visual complexity and highlight only the most meaningful influences, the total relation matrix (Τ) is filtered using a threshold value (ϱ). Several approaches for determining ϱ have been discussed by Si et al.,^40^ including setting it through expert judgment and discussion, calculating the average value of all entries in matrix (Τ), or using the maximum value among the diagonal elements of (Τ).

#### 2.3.8 Step 8. Attribute Weighting

The Prominence value reflects the extent to which attribute *j* occupies a central position within the decision problem. Accordingly, Prominence values can be employed to determine the relative importance of the attributes, as expressed in eq 19.

19 $$\omega_{j}=\frac{r_{j}+c_{j}}{\sum_{j=1}^{n}\left(r_{j}+c_{j}\right)}$$

## 3 PIV Method Extension under Picture Fuzzy Environment (PiF-PIV)

The proposed PiFN-based PIV method is illustrated in this section. The methodological contribution of this approach is that it introduces the first picture fuzzy set (PiFS)-based version of the PIV method, which can work with the unique data collection framework introduced in Section 2.2. Owing to the four-dimensional structure of the PiFS concept, this approach can be regarded as one of the most comprehensive extensions of fuzzy sets, as it captures a wider range of human judgments. Moreover, the unique data collection procedure, described in Steps 1.2 and 1.3 of the algorithm, removes the restrictions associated with predefined linguistic term sets. Instead of requiring experts to select from a conventional linguistic scale, the method directly collects their opinions in the form of YES, NO, ABSTAIN, or REFUSE. In this way, the need for a linguistic term set is eliminated.

### 3.1 Step 1. Decision Model

$\mathrm{\Gamma ̃}={\left[{\mathrm{\gamma ̃}}_{ij}\right]}_{m*n}$ presents the aggregated decision matrix where ${\mathrm{\gamma ̃}}_{ij}=(\alpha_{ij},\eta_{ij},\beta_{ij}$ ) is the aggregated performance assessments of alternative *i* with respect to attribute *j* (*i* = 1,···,*m*; *j* = 1,···,*n*). $W={\left[\omega_{j}\right]}_{1*n}$ is the weight vector.

### 3.2 Step 2. Data Gathering from Experts

Each alternative *i* is evaluated by each expert *E* by considering the alternative’s expected performance with respect to attribute *j*. In this evaluation, the expert E chooses the most appropriate linguistic terms among the set of YES, NO, ABSTAIN, and REFUSE. The details of this data collection scheme are explained in Section 2.2.

### 3.3 Step 3. Aggregation of the Expert Opinions

Section 2.2. Provides us with an approach to aggregate the opinions of experts. Equations 6–9) are performed for components of PiFNs, respectively. The aggregated decision matrix is presented by $\mathrm{\Gamma ̃}={\left[{\mathrm{\gamma ̃}}_{ij}\right]}_{m*n}$ .

### 3.4 Step 4. Weight Determination

In MADM, there are two main frameworks for attribute weighting. The subjective framework needs and processes expert opinions and judgments, while the objective one computes these weights by measuring some metrics from the current decision matrix. Since this study is designed from a subjective viewpoint, a subjective weighting framework is preferred. A PiF version of DEMATEL is selected for this purpose because of its extensive theoretical foundations studied in Section 2.3. The outcome of PiF-DEMATEL application is ω_*j*_.

### 3.5 Step 5. Computing Weighted Decision Matrix

Generally, in MADM, the decision matrix is first normalized. However, the data processing system performed by this study does not need any normalization step because all evaluations are based on expert judgments which are measured on the same scale. Therefore, the decision matrix is directly weighed without any normalization process.

The attribute weighting process is based on the scalar multiplication equation given in the sixth point of Definition 3. By performing eq 20, weighted decision matrix Υ = [Υ_*ij*_] is revealed where Υ_*ij*_ = (Α_*ij*_,Ν_*ij*_,Β_*ij*_).

20 $$W\mathrm{\Gamma ̃}=\left[\omega_{j}\times {\mathrm{\gamma ̃}}_{ij}\right]=\left[({Α}_{ij},{Ν}_{ij},{Β}_{ij})\right]=\left[(1-{\left(1-\alpha_{ij}\right)}^{\omega_{j}},{\left(\eta_{ij}\right)}^{\omega_{j}},{\left(\eta_{ij}+\beta_{ij}\right)}^{\omega_{j}}-{\left(\eta_{ij}\right)}^{\omega_{j}})\right]$$

### 3.6 Step 6. Weighted Proximity Index (WPI)

This measure is calculated for each Υ_*ij*_ and represents the deviation from the ideal, or best available, alternative, which is artificially constructed. To determine this ideal alternative, the best evaluation value is selected for each attribute, as shown in eq 21 which is produced from the first point of Definition 3. Α_*ij*_ includes the positive opinions (YES) so that maximum of Α_*ij*_ values should be selected. However, Β_*ij*_ and Ν_*ij*_ terms reflect a negative perspective because they include NO and ABSTAIN opinions, respectively. So, their minimum should be selected for the ideal solution.

21 $$\Upsilon_{best}=\left({Α}_{best},{Ν}_{best},{Β}_{best}\right)=\left(\max{Α}_{ij},\min{Ν}_{ij},\min{Β}_{ij}\right)$$

WPI_*ij*_ is the difference between the best solution (Υ_best_) and each alternative *i* for each attribute *j* (Υ_*ij*_). So, it indicates the extent to which an alternative *i* approaches the ideal solution for the corresponding attribute *j* (Eq. 22).

22 $${WPI}_{ij}=\Upsilon_{best}-\Upsilon_{ij}$$

To obtain this difference, the Euclidean distance measure for PiFNs as given in eq 23 is employed in this study.

23 $${WPI}_{ij}={\left[{\left({Α}_{best}-{Α}_{ij}\right)}^{2}+{\left({Ν}_{best}-{Ν}_{ij}\right)}^{2}+{\left({Β}_{best}-{Β}_{ij}\right)}^{2}\right]}^{1/2}$$

### 3.7 Step 7. Overall Proximity Index (OPI)

WPI_*ij*_ values are calculated for each alternative and attribute pair. The sum of them for each alternative *i* is called OPI_*i*_ (eq 24).

24 $${OPI}_{i}=\sum_{j=1}^{n}{WPI}_{ij}$$

OPI_*i*_ value indicates how closely an alternative resembles the best available option. Thus, the lower the OPI_*i*_ value, the preferable alternative *i* is. Accordingly, alternatives are ranked in ascending order based on their OPI_*i*_ values.

The applicability of the proposed PiF-DEMATEL-PIV method is illustrated in the following section through a plant extract selection problem which is based on the evaluations regarding their wound healing potentials. In addition, a sensitivity analysis is presented at the end to evaluate and demonstrate the robustness and reliability of the proposed algorithm.

## 4 Results: Ranking of Plant Extracts

The proposed MADM based method is applied to a real-world plant extract selection problem for obtaining better wound healing. Scientists, firms, product developers and healthcare professionals dealing with wound healing issue are in need of new materials and methods in order to boost wound healing efficiency. Not only the papers in the area, upon interactions with the aforementioned actors, authors are inspired by demands for the better performing wound healing materials and how plant extracts can be exploited to this aim.

In the selection of the plant extracts for this research, a multidimensional approach was considered, where the pharmacological appropriateness of the plant extracts for wound healing and their regional availability was both given high consideration. Certain specific botanical remedies were preferred in this research due to their well-documented properties of stimulating the regeneration of tissues and accelerating the healing process, which have been well established in previous phytotherapy and clinical studies. Apart from their specific healing properties, the regional abundance of these plants also played a significant role in the selection process.^42,43^ For example, certain plant extracts were preferred due to their ready availability in certain local climatic conditions or from common agricultural sources, which can be easily tapped and made economically feasible on a large scale for therapeutic purposes.^44,45^ Moreover, the selection process also took into consideration the knowledge and information gathered from expert discussions at international conferences and workshops, so that the selected plant extracts are in line with the current trends and demands of the pharmaceutical industry. In the selection process, the criteria went beyond the bioactive properties of the plant extracts to include environmental sustainability and regional economic feasibility, which is reflective of a multidimensional approach to material selection for the MADM analysis.

The present literature points out that why the selected plant extracts are of interest to researchers and medical community. Herbal extracts such as *C. asiatica* have many specific triterpenoids, which have been shown to enhance the expression of VEGF and TGF-beta. The pathway stimulates the migration of the endothelial cells to create new microvessels and supply oxygen to the hypoxic wound.^46^ Additionally, *Althaea officinalis* (*A. officinalis*) offers high molecular weight polysaccharides that create a physical barrier in addition to stimulation of epithelial cell proliferation in order to cover the wound edges.^47^ *Hypericum perforatum* and *Malva sylvestris* (*M. sylvestris*) have high contents of flavonoids, hyperforin, and anthocyanins, which work as strong scavengers of free radicals to inhibit Reactive Oxygen Species that may destroy the nascent matrix formed outside cells.^48^ Where bacteria of multidrug-resistant types complicate the healing of the wounds, *Tagetes erecta* (*T. erecta*) and *Achillea millefolium* (*A. millefolium*) have antibacterial properties that affect the microbial cell membranes. This facilitates biofilm inhibition and makes the transition from inflammation to proliferation easy for the wounds.^49^

Limitation in the current biomedical literature is that it is highly fragmented rather than pure focused. Although there are thousands of screenings in pharmacology where individual extracts of plants have been examined, these studies examine biological pathways individually. Rather than considering biochemical properties as isolated entities, this model considers the direct control exerted by secondary metabolites on cellular kinetics. It outlines the relationship between a plant’s chemical profile and its ability to perform in terms of tissue repair.^50,51^ Those plant-based chemicals with the highest medicinal speed are those with the lowest toxicity threshold.^52^ The incorporation of an expert panel with both a background in clinical phytotherapy and materials science allows this framework to identify the “optimal compromise” between the two extremes of regeneration and biological safety.^53,54^ Through the incorporation of sustainability and availability alongside biological properties, the current model ensures that the selected plant candidates can be industrially processed (for example through electrospinning into dressings).

Our aim is to evaluate and compare the wound-healing potential of plant extracts using MADM methods, with the goal of identifying the most effective formulation for promoting tissue regeneration and accelerating recovery.

### 4.1 PiF-DEMATEL for Attribute Weighting

PiF extension of DEMATEL is applied to obtain the importance of attributes that will be represented by weights. The details are as follows.

### 4.2 Step 1. Determining the Attributes and the Experts

This study is designed as a preliminary tool to provide researchers with enriched information on the potential of plant extracts before any experiments. Five experts (*e* = 1, ..., *E* = *5*) are contacted for their knowledge and expertise regarding the research topic. The details of these experts’ background are presented in Table 1.

**Table 1:** Background of Experts

E	expert description	institution/country
*e*_1_	DM1 is an Associate Professor specializing in plant-derived bioactives and antimicrobial functional materials, with a publication track record focused on phytochemical extracts and essential oils assessed in terms of measurable biological performance. Her research work again and again targets wound-associated pathogens such as Pseudomonas aeruginosa and Staphylococcus spp. and demonstrates antiquorum sensing, antibiofilm, and antibacterial properties of various botanicals (*palmarosa, niaouli, Opuntia, Prunus, Persea*), sometimes in combination with cytocompatibility measures such as fibroblast viability. She also employs in silico approaches (DFT calculations, docking) for mechanism-based comparisons and is engaged in green nanotechnology, including plant-mediated biosynthesis of Ag and ZnO nanoparticles with biomedical activity profiles and encapsulation-based antibacterial formulations. In the context of “Comparison of Wound Healing Potential of Plant Extracts with MADM Methods,” her evidence-creation capability facilitates effective criteria definition and data entry for hybrid MADM ranking, thus helping to make an objective choice of promising and sustainable herbal remedies	Faculty of pharmacy, Süleyman Demirel University/Türkiye
*e*_2_	DM2 is a researcher in pharmaceutical botany with a 2025 graduate thesis on the botanical and pharmaceutically significant study of Origanum solymicum P.H. Davis and Origanum saccatum P.H. Davis. In a 2025 peer-reviewed article, he also reported herbal drugs marketed in herbal shops in Turkey’s Western Mediterranean area for circulatory system diseases, giving evidence on real-world usage patterns and sources of plant-based products. In “Comparison of Wound Healing Potential of Plant Extracts with MADM Methods,” this expertise is relevant to the study by improving upstream choices: proper species identification, quality and authenticity of raw materials, and the incorporation of ethnobotanical/marketevidence into criteria such as availability, standardization feasibility, and sustainability—to help ensure that MADM rankings not only measure bioactivity, but also the potential for clinical translation	Faculty of Pharmacy, Süleyman Demirel University/Türkiye
*e*_3_	DM3 is a phytochemist and pharmaceutical botanist & Assistant Professor whose research output exactly matches the requirements for “Comparison of Wound Healing Potential of Plant Extracts with MADM Methods.” A highly cited research stream is the comparative analysis of Turkish medicinal plants (especially Achillea species), correlating phenolic content with antioxidant activity, wound healing, and cytotoxicity in a single study design, which is optimal for multicriteria analysis. His research interests also include advanced chemical analysis (such as LC-ESI-QTOF-MS/MS phenolic analysis), method validation (HPLC method development), and pharmacopoeial standardization (such as Rosa damascena), which enhance criteria such as extract quality, reproducibility, and readiness for translation. In a hybrid MADM environment, his research background can help with criteria definition and scoring in a bioactivity, safety, standardization feasibility, and sustainability context, facilitating more objective selection of plant materials for next-generation wound healing remedies	Faculty of Pharmacy, Süleyman Demirel University/Türkiye
*e*_4_	DM4 is a materials scientist and nanotechnology researcher whose research output has excellent evidence-generation potential for “Comparison of Wound Healing Potential of Plant Extracts with MADM Methods.” Her research focus is on green, plant extract-mediated synthesis of nanoparticles such as Ag, ZnO, Au, and Ag–Au core–shell nanoparticles, and the assessment of their antibacterial, antiproliferative, cytotoxicity, and biocompatibility properties. She has also contributed to the development of fiber-based biomedical and functional materials such as electrospun PLA fibers doped with plant-synthesized AgNPs, and has extensive process know-how in polymer microfibers and textile-based filtration media, which will aid in the reproducibility and scale-appropriate evaluation. For a hybrid MADM approach, her expertise can be leveraged to transform plant extracts into comparable and quantifiable criteria such as extraction process and phenolic content, antimicrobial efficacy, safety for healthy cells, and processing feasibility (nanoparticle incorporation and fiber production). This will enhance the objective prioritization and short-listing of plant species that are not only biologically effective but also economically and environmentally viable for the development of next-generation wound healing technologies	Faculty of Pharmacy, Süleyman Demirel University/Türkiye
e_5_	DM5 is a pharmacist-scientist focused on traditional herbal medicines and bioactive phytoconstituents, with a research record that turns ethnobotanical leads into experimentally validated therapeutic promise. Her publications include highly cited work with direct relevance to wound care—for example, studies reporting that purified constituents from Plantago species inhibit collagenase, elastase, and hyaluronidase, enzymes linked to extracellular matrix breakdown and delayed wound healing. She has also added to the evidence base on essential oils and plant extracts with wound-repair or infection-related mechanisms, alongside broader expertise in anti-inflammatory and antimicrobial/antiquorum sensing assessments of medicinal plants. Beyond academia, she brings hands-on leadership experience as the owner of an independent community pharmacy and as an active member of pharmacy chambers, giving her a strong perspective on real-world feasibility, patient safety, and adoption constraints. For “Comparison of Wound Healing Potential of Plant Extracts with MADM Methods,” her profile supports rigorous criteria selection and balanced evidence interpretation across mechanistic strength, antimicrobial relevance, safety, and translational practicality—strengthening an objective hybrid MCDM ranking of herbal candidates for sustainable next-generation wound care.	pharmacy owner/Türkiye

This study aims at assessing the potential of wound-healing plant extracts by considering multiple attributes. The alternatives are scored by five experts with respect to these 8 attributes (*j* = 1,···,*n* = 8). Their definitions, significance, and proper references are introduced as follows:

J_1_. Cell regeneration: the process by which cells proliferate, differentiate, and transdifferentiate to replace or repair damaged tissues is known as cell regeneration. This process is impacted by a number of cellular and microenvironmental factors.^55,56^

J_2_. Angiogenic activity: angiogenesis, the act of creating new blood vessels from preexisting ones, is crucial to the healing of wounds. In addition to supplying nourishment and oxygen to developing tissues, newly created blood vessels take part in the creation of temporary granulation tissue^57,58^

J_3_. Anti-inflammatory effect: the term “anti-inflammatory” describes drugs or molecules that lessen the production of inflammatory mediators or prevent the release of histamine, therefore reducing inflammatory conditions.^59,60^

J_4._ Phytochemical content: non-nutritive plant compounds known as phytochemicals are the secondary plant metabolites and have the ability to prevent or treat disease. Phytochemical content is the quantity and range of naturally occurring bioactive chemical substances that plants produce.^61,62^

J_5_. Antimicrobial activity: the ability of a material or medication to prevent or eradicate the growth and reproduction of bacteria is known as antimicrobial activity.^51,63^

J_6_. Antioxidant activity: antioxidant activity refers to an antioxidant’s overall ability to remove free radicals from food and cells. It is a complex process that relies on each antioxidant’s specific role.^64,65^

J_7_. Abundancy: the availability, distribution, and accessibility of a particular plant species in nature or through cultivation are all considered aspects of abundance when discussing plants used for wound healing. It indicates how easily a plant can be obtained in order to extract its bioactive components and use them into applications such as medicine or biomaterials.^66,67^

J_8_. Toxicity: a substance’s harmful effects on a living thing are determined by how much of it is given or absorbed, how it is administered, and other factors.^68,69^

DEMATEL requests the judgments of the experts regarding the influences among attributes. The unique data gathering system, which is introduced in Section 2.2, is utilized in this PiF-extended DEMATEL implementation. Each expert’s individual evaluations are presented in Appendix A.

### 4.3 Step 2. Obtaining Direct PiF Influence Matrix.

The expert judgment for each attribute pair is defined with only one linguistic term of YES­(the agreement level), NO, ABSTAIN, or REFUSE. The direct PiF influence matrix $\mathrm{\Psi ̃}={\left[{\mathrm{\gamma ̃}}_{jk}\right]}_{8*8}$ (Table 2) is obtained by performing eqs 10–13).

**Table 2:** Direct Influence Matrix of PiF-DEMATEL

	*j* = 1				*j* = 2				*j* = 3				*j* = 4			
	yes	abstain	no	refuse	yes	abstain	no	refuse	yes	abstain	no	refuse	yes	abstain	no	refuse
*j* = 1	1.000	0.000	0.000	0.000	0.760	0.120	0.000	0.120	0.680	0.160	0.000	0.160	0.450	0.075	0.400	0.075
*j* = 2	0.810	0.095	0.000	0.095	1.000	0.000	0.000	0.000	0.550	0.225	0.000	0.225	0.400	0.100	0.400	0.100
*j* = 3	0.760	0.120	0.000	0.120	0.590	0.205	0.000	0.205	1.000	0.000	0.000	0.000	0.430	0.085	0.400	0.085
*j* = 4	0.740	0.130	0.000	0.130	0.500	0.250	0.000	0.250	0.740	0.130	0.000	0.130	1.000	0.000	0.000	0.000
*j* = 5	0.640	0.180	0.000	0.180	0.350	0.225	0.200	0.225	0.520	0.240	0.000	0.240	0.720	0.140	0.000	0.140
*j* = 6	0.700	0.150	0.000	0.150	0.570	0.215	0.000	0.215	0.670	0.165	0.000	0.165	0.820	0.090	0.000	0.090
*j* = 7	0.000	0.000	1.000	0.000	0.000	0.000	1.000	0.000	0.000	0.000	1.000	0.000	0.000	0.000	1.000	0.000
*j* = 8	0.640	0.180	0.000	0.180	0.480	0.260	0.000	0.260	0.510	0.245	0.000	0.245	0.400	0.300	0.000	0.300

	*j* = 5				*j* = 6				*j* = 7				*j* = 8			
	yes	abstain	no	refuse	yes	abstain	no	refuse	yes	abstain	no	refuse	yes	abstain	no	refuse
*j* = 1	0.380	0.310	0.200	0.110	0.500	0.250	0.200	0.050	0.000	0.000	1.000	0.000	0.260	0.170	0.400	0.170
*j* = 2	0.090	0.255	0.600	0.055	0.220	0.290	0.400	0.090	0.040	0.080	0.800	0.080	0.180	0.210	0.400	0.210
*j* = 3	0.480	0.260	0.000	0.260	0.590	0.205	0.000	0.205	0.000	0.000	1.000	0.000	0.260	0.270	0.200	0.270
*j* = 4	0.740	0.130	0.000	0.130	0.840	0.080	0.000	0.080	0.140	0.330	0.800	0.330	0.320	0.240	0.200	0.240
*j* = 5	1.000	0.000	0.000	0.000	0.440	0.180	0.200	0.180	0.080	0.360	0.800	0.360	0.300	0.150	0.000	0.150
*j* = 6	0.400	0.200	0.200	0.200	1.000	0.000	0.000	0.000	0.100	0.350	0.800	0.350	0.260	0.470	0.000	0.270
*j* = 7	0.000	0.000	1.000	0.000	0.000	0.000	1.000	0.000	1.000	0.000	0.000	0.000	0.000	0.000	1.000	0.000
*j* = 8	0.440	0.280	0.000	0.280	0.320	0.340	0.000	0.340	0.000	0.000	1.000	0.000	1.000	0.000	0.000	0.000

### 4.4 Step 3. Obtaining Direct Influence Matrix

Since there are many matrix operations involving inversion and matrix multiplications, PiF numbers presented in Table 2 are defuzzified via eq 14. Table 3 depicts the defuzzified direct influence matrix. As an example, the details of defuzzifying (α_12_,η_12_,β_12_) = (0.760, 0.120, 0.000) is given below.

$$\gamma_{12}=0.760+\frac{0.120}{2}+\frac{1-0.760-0.120-0.000}{2}\left(1+0.760-0.000\right)=0.926$$

**Table 3:** Defuzzified Direct Influence Matrix of PiF-DEMATEL

	*j* = 1	*j* = 2	*j* = 3	*j* = 4	*j* = 5	*j* = 6	*j* = 7	*j* = 8	
*j* = 1	1.000	0.926	0.894	0.527	0.600	0.658	0.000	0.418	5.022
*j* = 2	0.943	1.000	0.837	0.500	0.231	0.402	0.090	0.367	4.370
*j* = 3	0.926	0.855	1.000	0.516	0.802	0.855	0.000	0.538	5.493
*j* = 4	0.918	0.813	0.918	1.000	0.918	0.954	0.259	0.574	6.354
*j* = 5	0.878	0.592	0.822	0.910	1.000	0.642	0.226	0.733	5.803
*j* = 6	0.903	0.846	0.890	0.947	0.620	1.000	0.238	0.665	6.109
*j* = 7	0.000	0.000	0.000	0.000	0.000	0.000	1.000	0.000	1.000
*j* = 8	0.878	0.802	0.817	0.760	0.782	0.714	0.000	1.000	5.753
	6.445	5.834	6.180	5.160	4.953	5.224	1.813	4.295	*k* = 6.445

The resulting Ψ = [**γ**_**jk**_] direct influence matrix including defuzzified values is presented in Table 3.

### 4.5 Step 4. Building Initial Direct Influence Matrix

Ψ is converted to Ζ = [ε_jk_] via executing eq 15. Row and column maximums are shown in the last row and column of Table 3, respectively, and the normalization index *k* is obtained as 6.445. Table 4 presents Ζ = [ε_jk_].

$$k=\max\left[\max_{j}\left(6.445,5.834,...,4.295\right),\max_{k}\left(5.022,4.370,...,5.753\right)\right]=\max\left[6.445,6.354\right]=6.445$$

**Table 4:** Initial Direct Influence Matrix of PiF-DEMATEL

	*j* = 1	*j* = 2	*j* = 3	*j* = 4	*j* = 5	*j* = 6	*j* = 7	*j* = 8
*j* = 1	0.155	0.144	0.139	0.082	0.093	0.102	0.000	0.065
*j* = 2	0.146	0.155	0.130	0.078	0.036	0.062	0.014	0.057
*j* = 3	0.144	0.133	0.155	0.080	0.125	0.133	0.000	0.083
*j* = 4	0.142	0.126	0.142	0.155	0.142	0.148	0.040	0.089
*j* = 5	0.136	0.092	0.128	0.141	0.155	0.100	0.035	0.114
*j* = 6	0.140	0.131	0.138	0.147	0.096	0.155	0.037	0.103
*j* = 7	0.000	0.000	0.000	0.000	0.000	0.000	0.155	0.000
*j* = 8	0.136	0.125	0.127	0.118	0.121	0.111	0.000	0.155

### 4.6 Step 5. Determining Total Influence Matrix

Equation 16 reveals the total influence matrix, Τ = [τ_jk_], as given in Table 5. The details of Ζ­(*I* – Ζ)^−1^ is skipped in order not to interrupt the continuity flow of the text.

**Table 5:** Total Influence Matrix of PiF-DEMATEL

	*j* = 1	*j* = 2	*j* = 3	*j* = 4	*j* = 5	*j* = 6	*j* = 7	*j* = 8
*j* = 1	0.833	0.762	0.789	0.602	0.596	0.642	0.094	0.492
*j* = 2	0.716	0.677	0.676	0.510	0.453	0.513	0.093	0.412
*j* = 3	0.892	0.815	0.873	0.658	0.683	0.731	0.105	0.559
*j* = 4	0.984	0.891	0.950	0.811	0.775	0.824	0.169	0.625
*j* = 5	0.909	0.793	0.869	0.745	0.741	0.720	0.152	0.610
*j* = 6	0.949	0.869	0.914	0.776	0.701	0.806	0.159	0.619
*j* = 7	0.000	0.000	0.000	0.000	0.000	0.000	0.184	0.000
*j* = 8	0.927	0.845	0.885	0.733	0.716	0.744	0.111	0.664

### 4.7 Step 6. Computing Prominence and Relation Values

The calculation details for the rest of the algorithm are presented in Table 6. First, the row sum (*R*) and column sum (*C*) are obtained through Eqs. 17–18) for attributes. Then, Prominence (*R* + *C*) and Relation (*R* – *C*), which are the components of IRM, are computed. The sign of the Relation values indicates the group of the attribute.

**Table 6:** PiF-DEMATEL Results

	*R*	*C*	*R* – *C*	group	*R* + *C*	∑(*R* + *C*)	ω	RANK
*j* = 1	4.810	6.210	–1.400	effect	11.020	11.108	0.147	2
*j* = 2	4.051	5.652	–1.601	effect	9.704	9.835	0.130	6
*j* = 3	5.317	5.956	–0.639	effect	11.273	11.291	0.150	1
*j* = 4	6.030	4.834	1.195	cause	10.864	10.930	0.145	3
*j* = 5	5.539	4.665	0.874	cause	10.205	10.242	0.136	5
*j* = 6	5.792	4.981	0.811	cause	10.774	10.804	0.143	4
*j* = 7	0.184	1.067	–0.884	effect	1.251	1.531	0.020	8
*j* = 8	5.624	3.981	1.643	cause	9.605	9.745	0.129	7

### 4.8 Step 7. Drawing Influential Relation Map (IRM)

The Prominence values are placed on the horizontal axis, and the Relation values are shown on the vertical axis. Therefore, Cause group attributes are located in the upper part of the IRM, whereas Effect group attributes are shown in the lower part. Figure 1 depicts the resulting IRM.

**Figure 1:**
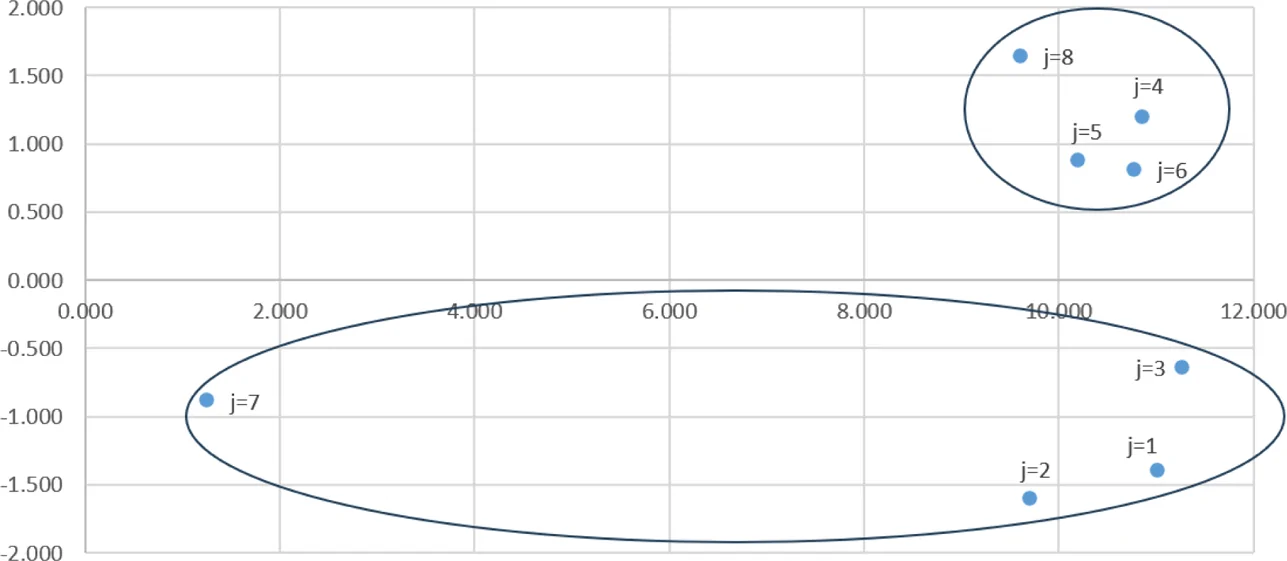
IRM

The two domains of criteria as illustrated in Figure 1 include the upstream biochemical drivers on top and the downstream physiological responders at the bottom. The top cluster includes the four parameters of phytochemical content (*j* = 4), antimicrobial activity (*j* = 5), antioxidant activity (*j* = 6), and toxicity (*j* = 8). The close proximity of *j* = 4, 5, 6 proves that diversity of secondary metabolites determines the chemical potency. At the same time, the clustering of toxicity (*j* = 8) means that metabolic potency and toxicity in vitro are related intrinsically. On the other hand, the three criteria at the bottom form a cluster of physiological responders including cell regeneration (*j* = 1), angiogenic activity (*j* = 2), and anti-inflammatory effects (*j* = 3). Instead of being independent variables, the listed characteristics define in vivo development of the process and depend on optimization of the upstream factors. Thus, increasing the parameters of antioxidant activity (*j* = 6) and phytochemical content (*j* = 4) help to reduce hyper-inflammation (*j* = 3). Increasing the level of antimicrobial activity (*j* = 5) helps to get rid of pathogens, which indirectly accelerates cell regeneration (*j* = 1) and leads to angiogenesis (*j* = 2). At last, abundancy (*j* = 7) serves as an outstanding *X*-axis criterion in the lower domain. While the previous six characteristics are performance-related, the criterion is related to logistic aspects of the work.

### 4.9 Step 8. Attribute Weighting

The Prominence values are normalized via eq 19, and the attribute weights are revealed. The weights and their rankings are given in the last two columns of Table 6. Accordingly, anti-inflammatory effect (ω_3_ = 0.150) ranked first. This is the most crucial physiological point of access to healing. The resolution of the condition of hyper-inflammation is a precondition for all the following phases. Cell regeneration (ω_1_ = 0.147) ranked second. This is the most important goal of wound healing from the clinical point of view. Phytochemical content (ω_4_ = 0.145) ranked third but classified as ″Cause″ with maximum net effect. This is the basic biochemical engine of the extract. A great variety of phytochemicals is necessary to neutralize any threats and cause the physiological effects described above. The relevance of these three criteria shows that the strength of the developed model lies in the mathematics that prove that in order to attain the most desirable outcomes in terms of clinical success and cellular regeneration, biopharmaceuticalists have to focus on phytochemical-based extracts.

### 4.10 PiF-DEMATEL for Attribute Weighting

The theoretical contribution of this study is based on a proposition of PiF extension of PIV that can work with the data gathered by utilizing the unique data collection system. The application of PiF-PIV is shown in detail as follows.

#### 4.10.1 Step 1. Decision Model

This study considers 10 materials (*i* = 1, ..., *m* = 10) for assessing their potential wound-healing capacity via an expert judgment-based evaluation system. The alternatives considered are listed below. The attributes (*j* = 1, ..., *n* = 8) and the experts (*e* = 1, ..., *E* = 5) are previously introduced in Section 4.1.

I_1_. *A. millefolium*: traditionally called yarrow, *A. millefolium* L. is a flowering plant belongs to the family *Asteraceae* that is widely utilized in folk medicine in Asia, Africa, and America in addition to Europe.^70,71^

I_2_. *Plantago major*: *P. major* (P. major) is a perennial plant which belongs to the Plantaginaceae family and its leaves have traditionally been used to heal wounds.^72,73^

I_3_. *H. perforatum*: *H. perforatum* belongs to the Hypericaceae family and is one of the oldest used and most extensively investigated medicinal herbs. It offers antidepressant, wound-healing, antioxidant, antiviral, and analgesic properties for burns, bruising, and edema.^74,75^

I_4_. *M. sylvestris*: *M. sylvestris* is a flowering plant belonging to the *Malvaceae* family that thrives in Europe, Asia, and North Africa and is characterized for its anti-inflammatory and antioxidant activity. It is also referred to names such as mellow, vilayatti or gulkhaira, kangan, marva, ebegümeci and khabazi in different countries.^76,77^

I_5_. *A. officinalis*: *A. officinalis*, commonly known as marshmallow, belongs to the Malvaceae plant family and has long history of uses as a food and folk medicinal plant. It contains bioactive compounds such as flavonoids and phenolics that support wound healing.^78,79^

I_6_. *C. asiatica*: *C. asiatica*, a perennial plant from the *Apiaceae* family. It is a plant with a history in traditional medicine, has particularly shown promise in the management of diabetic complications.^80,81^

I_7_. *Calendula officinalis*: The Asteraceae family includes the blooming plant known as *C. officinalis*, sometimes referred to as pot marigold, which exhibits anti-inflammatory properties.^82,83^

I_8_. *Momordica charantia*: *M. charantia* (M. charantia), commonly referred to as bitter melon, bitter gourd, karela, or balsam pear, is a member of the Cucurbitaceae family and it is recognized for its therapeutic properties attributed to the abundance of its bioactive constituents.^84,85^

I_9_. *T. erecta*: *T. erecta* (Marigold) is an endemic Mexican species belongs to the Asteraceae family. It is well-known for its antibacterial, anti-inflammatory, and antioxidant activities, as well as wound-healing effects.^86,87^

I_10_. *Matricaria recutita*: *M. recutita* (M. recutita), which belongs to the Asteraceae family, is known as chamomile and has been studied for years because of its agronomic and phytochemical aspects.^88,89^

#### 4.10.2 Step 2. Data Gathering from Experts

Utilizing the PiF-unique data collection approach, each alternative *i* is scored with respect to each attribute *j* by each expert *e*. The algorithm of this data collection system is detailed in Section 2.2. Each expert’s individual evaluations on alternatives are presented in Appendix B.

#### 4.10.3 Step 3. Aggregation of the Expert Opinions

Five different evaluations are combined by using eqs 6–9) and the aggregated decision matrix (Table 7) is built ( $\mathrm{\Gamma ̃}={\left[{\mathrm{\gamma ̃}}_{ij}\right]}_{10*8}$ ).

**Table 7:** Aggregated Decision Matrix of PiF-DEMATEL

	*j* = 1				*j* = 2				*j* = 3				*j* = 4			
	yes	abstain	no	refuse	yes	abstain	no	refuse	yes	abstain	no	refuse	yes	abstain	no	refuse
*i* = 1	0.710	0.145	0.000	0.145	0.550	0.225	0.000	0.225	0.800	0.100	0.000	0.100	0.870	0.065	0.000	0.065
*i* = 2	0.710	0.145	0.000	0.145	0.460	0.270	0.000	0.270	0.780	0.110	0.000	0.110	0.850	0.075	0.000	0.075
*i* = 3	0.830	0.085	0.000	0.085	0.690	0.155	0.000	0.155	0.800	0.100	0.000	0.100	0.910	0.045	0.000	0.045
*i* = 4	0.660	0.170	0.000	0.170	0.490	0.255	0.000	0.255	0.770	0.115	0.000	0.115	0.800	0.100	0.000	0.100
*i* = 5	0.670	0.165	0.000	0.165	0.450	0.275	0.000	0.275	0.720	0.140	0.000	0.140	0.770	0.115	0.000	0.115
*i* = 6	0.916	0.042	0.000	0.042	0.830	0.085	0.000	0.085	0.820	0.090	0.000	0.090	0.920	0.040	0.000	0.040
*i* = 7	0.800	0.100	0.000	0.100	0.700	0.150	0.000	0.150	0.810	0.095	0.000	0.095	0.800	0.100	0.000	0.100
*i* = 8	0.690	0.155	0.000	0.155	0.570	0.215	0.000	0.215	0.690	0.155	0.000	0.155	0.760	0.120	0.000	0.120
*i* = 9	0.580	0.210	0.000	0.210	0.510	0.245	0.000	0.245	0.600	0.200	0.000	0.200	0.710	0.145	0.000	0.145
*i* = 10	0.700	0.150	0.000	0.150	0.590	0.205	0.000	0.205	0.830	0.085	0.000	0.085	0.830	0.085	0.000	0.085

	*j* = 5				*j* = 6				*j* = 7				*j* = 8			
	yes	abstain	no	refuse	yes	abstain	no	refuse	yes	abstain	no	refuse	yes	abstain	no	refuse
*i* = 1	0.660	0.170	0.000	0.170	0.800	0.100	0.000	0.100	0.800	0.100	0.000	0.100	0.156	0.422	0.000	0.422
*i* = 2	0.620	0.190	0.000	0.190	0.750	0.125	0.000	0.125	0.870	0.065	0.000	0.065	0.086	0.457	0.000	0.457
*i* = 3	0.760	0.120	0.000	0.120	0.800	0.100	0.000	0.100	0.780	0.110	0.000	0.110	0.340	0.330	0.000	0.330
*i* = 4	0.550	0.225	0.000	0.225	0.770	0.115	0.000	0.115	0.800	0.100	0.000	0.100	0.100	0.450	0.000	0.450
*i* = 5	0.440	0.280	0.000	0.280	0.630	0.185	0.000	0.185	0.720	0.140	0.000	0.140	0.070	0.465	0.000	0.465
*i* = 6	0.610	0.195	0.000	0.195	0.780	0.110	0.000	0.110	0.660	0.170	0.000	0.170	0.150	0.425	0.000	0.425
*i* = 7	0.660	0.170	0.000	0.170	0.760	0.120	0.000	0.120	0.780	0.110	0.000	0.110	0.134	0.433	0.000	0.433
*i* = 8	0.600	0.200	0.000	0.200	0.720	0.140	0.000	0.140	0.700	0.150	0.000	0.150	0.300	0.350	0.000	0.350
*i* = 9	0.630	0.185	0.000	0.185	0.660	0.170	0.000	0.170	0.660	0.170	0.000	0.170	0.160	0.420	0.000	0.420
*i* = 10	0.610	0.195	0.000	0.195	0.770	0.115	0.000	0.115	0.700	0.150	0.000	0.150	0.156	0.422	0.000	0.422

#### 4.10.4 Step 4. Weight Determination

PiF-DEMATEL reveals the influences among attributes and obtains the attribute weights (ω_*j*_) which are based on these influences. The attribute weights are seen in Table 6.

#### 4.10.5 Step 5. Computing Weighted Decision Matrix

Equation 20 produces the weighted decision matrix Υ = [Υ_*ij*_] where Υ_*ij*_ = (Α_*ij*_,Ν_*ij*_,Β_*ij*_). Table 8 depicts the matrix Υ. As an example, the weighted evaluation of the first alternative with respect to the second attribute is computed as follows

$$\left({Α}_{12},{Ν}_{12},{Β}_{12}\right)=\omega_{2}{\tilde{\times \gamma }}_{12}=0.130\times \left(0.550,0.225,0\right)$$

$$=\left[(1-{\left(1-0.550\right)}^{0.130},{\left(0.225\right)}^{0.130},{\left(0.225+0\right)}^{0.130}-0.225^{0.130})\right]$$

=(0.099,0.823,0)

**Table 8:** Weighted Decision Matrix of PiF-DEMATEL

	*j* = 1				*j* = 2				*j* = 3				*j* = 4			
	yes	abstain	no	refuse	yes	abstain	no	refuse	yes	abstain	no	refuse	yes	abstain	no	refuse
*i* = 1	0.167	0.753	0.000	0.081	0.099	0.823	0.000	0.078	0.214	0.709	0.000	0.077	0.256	0.673	0.000	0.071
*i* = 2	0.167	0.753	0.000	0.081	0.077	0.843	0.000	0.080	0.203	0.719	0.000	0.079	0.240	0.687	0.000	0.073
*i* = 3	0.230	0.696	0.000	0.075	0.142	0.784	0.000	0.074	0.214	0.709	0.000	0.077	0.294	0.638	0.000	0.067
*i* = 4	0.147	0.770	0.000	0.083	0.084	0.837	0.000	0.079	0.197	0.724	0.000	0.079	0.208	0.716	0.000	0.076
*i* = 5	0.151	0.767	0.000	0.082	0.075	0.845	0.000	0.080	0.173	0.745	0.000	0.081	0.192	0.731	0.000	0.077
*i* = 6	0.305	0.627	0.000	0.067	0.206	0.725	0.000	0.069	0.226	0.698	0.000	0.076	0.306	0.627	0.000	0.066
*i* = 7	0.211	0.713	0.000	0.077	0.145	0.781	0.000	0.074	0.220	0.703	0.000	0.077	0.208	0.716	0.000	0.076
*i* = 8	0.158	0.760	0.000	0.082	0.104	0.819	0.000	0.077	0.161	0.757	0.000	0.083	0.187	0.736	0.000	0.078
*i* = 9	0.120	0.795	0.000	0.085	0.089	0.833	0.000	0.079	0.128	0.786	0.000	0.086	0.164	0.756	0.000	0.080
*i* = 10	0.162	0.756	0.000	0.081	0.110	0.813	0.000	0.077	0.233	0.692	0.000	0.076	0.226	0.700	0.000	0.074
best	0.305	0.627	0.000	0.067	0.206	0.725	0.000	0.069	0.233	0.692	0.000	0.076	0.306	0.627	0.000	0.066

	*j* = 5				*j* = 6				*j* = 7				*j* = 8			
	yes	abstain	no	refuse	yes	abstain	no	refuse	yes	abstain	no	refuse	yes	abstain	no	refuse
*i* = 1	0.136	0.786	0.000	0.078	0.206	0.719	0.000	0.075	0.032	0.954	0.000	0.014	0.022	0.895	0.000	0.084
*i* = 2	0.123	0.798	0.000	0.079	0.180	0.743	0.000	0.077	0.041	0.946	0.000	0.013	0.012	0.904	0.000	0.085
*i* = 3	0.176	0.750	0.000	0.074	0.206	0.719	0.000	0.075	0.030	0.956	0.000	0.014	0.052	0.867	0.000	0.081
*i* = 4	0.103	0.817	0.000	0.081	0.190	0.734	0.000	0.077	0.032	0.954	0.000	0.014	0.014	0.902	0.000	0.084
*i* = 5	0.076	0.841	0.000	0.083	0.133	0.785	0.000	0.082	0.025	0.961	0.000	0.014	0.009	0.906	0.000	0.085
*i* = 6	0.120	0.801	0.000	0.079	0.195	0.729	0.000	0.076	0.022	0.965	0.000	0.014	0.021	0.895	0.000	0.084
*i* = 7	0.136	0.786	0.000	0.078	0.185	0.738	0.000	0.077	0.030	0.956	0.000	0.014	0.018	0.898	0.000	0.084
*i* = 8	0.117	0.804	0.000	0.079	0.167	0.755	0.000	0.079	0.024	0.962	0.000	0.014	0.045	0.873	0.000	0.082
*i* = 9	0.126	0.795	0.000	0.078	0.143	0.776	0.000	0.081	0.022	0.965	0.000	0.014	0.022	0.894	0.000	0.084
*i* = 10	0.120	0.801	0.000	0.079	0.190	0.734	0.000	0.077	0.024	0.962	0.000	0.014	0.022	0.895	0.000	0.084
best	0.176	0.750	0.000	0.074	0.206	0.719	0.000	0.075	0.041	0.946	0.000	0.013	0.052	0.867	0.000	0.081

#### 4.10.6 Step 6. Weighted Proximity Index (WPI)

In order to create the evaluation score, PIV first needs the ideal (best) solution. Equation 21 is implemented for this purpose, and the obtained artificial ideal solution is shown in the row called best of Table 8. Then, the distance between each alternative and this ideal solution is measured via eq 22, which is based on eq 23. WPI_*ij*_ values are shown in Table 9. WPI_12_ is computed as follows

$${WPI}_{12}=d\left[\left(0.206,0.725,0\right),\left(0.099,0.823,0\right)\right]$$

$$={\left[{\left(0.206-0.099\right)}^{2}+{\left(0.725-0.823\right)}^{2}+{\left(0-0\right)}^{2}\right]}^{1/2}=0.145$$

**Table 9:** Findings of PiF-PIV

	WPI_*ij*_									
	*j* = 1	*j* = 2	*j* = 3	*j* = 4	*j* = 5	*j* = 6	*j* = 7	*j* = 8	OPI_i_	rank
*i* = 1	0.187	0.145	0.025	0.068	0.054	0.000	0.012	0.041	0.533	4
*i* = 2	0.187	0.175	0.041	0.089	0.072	0.035	0.000	0.055	0.653	6
*i* = 3	0.102	0.088	0.025	0.016	0.000	0.000	0.014	0.000	0.246	2
*i* = 4	0.214	0.165	0.048	0.133	0.099	0.022	0.012	0.052	0.745	7
*i* = 5	0.209	0.178	0.080	0.155	0.136	0.099	0.021	0.058	0.935	9
*i* = 6	0.000	0.000	0.009	0.000	0.076	0.015	0.027	0.043	0.169	1
*i* = 7	0.127	0.083	0.017	0.133	0.054	0.028	0.014	0.046	0.503	3
*i* = 8	0.198	0.138	0.097	0.161	0.080	0.053	0.023	0.010	0.761	8
*i* = 9	0.250	0.159	0.141	0.192	0.067	0.085	0.027	0.041	0.961	10
*i* = 10	0.193	0.131	0.000	0.108	0.076	0.022	0.023	0.041	0.593	5

#### 4.10.7 Step 7. Overall Proximity Index (OPI)

The row sums of WPI_*ij*_ provides OPI_*i*_ (eq 24) which is the overall distance of each alternative from the ideal one. The lower OPI_*i*_, the closer to ideal. Table 9 reports on these findings. According to these results, *C. Asiatica* is found as the best plant extract for wound-healing and is followed by *H. Perforatum* and *Calendula Officinalis*. The full ranking of alternatives can be observed in the last column of Table 8. The results are discussed in Section 5.

### 4.11 Robustness Check

The common and generally accepted final step in MADM applications is to check the robustness of the alternatives against changing weighting and/or ranking methods. For this purpose, we designed eight different settings in addition to the original PiF-DEMATEL-PIV concept. In these experiments, we adopted entropy-based weighting and CRITIC weighting for attributes, and TOPSIS and ARAS for alternative rankings.^41^

- Entropy-based attribute weighting just considers the internal distribution of the data set for each attribute and assigns the weights according to the magnitude of these distributions. The distribution level is measured by Shannon’s entropy definition. The higher the divergence, the higher the importance.
- CRITIC weighting system is based on both internal distributions which are measured by standard deviation or entropy and the external relationships among attributes which are measured by correlation coefficient. These two measures are blended, and the attribute weights are revealed.
- TOPSIS ranks alternatives according to their closeness to ideal solutions that represent the most favorable and least favorable outcomes across all criteria. The ideal solution is a hypothetical benchmark that may not exist in practice but serves as a target for comparison. In TOPSIS, alternatives are evaluated by calculating their Euclidean distances from both the positive ideal solution and the negative ideal solution. The preferred alternative is the one that is nearest to the positive ideal solution while being farthest from the negative ideal solution.
- ARAS ranks the alternatives by comparing their normalized and weighted attribute-wise evaluations with an optimal solution which is designed as we do in TOPSIS. The optimal solution is built by selecting the best score included by each attribute. Then, the aggregated scores of alternatives and this optimal solution are compared as a ratio. Finally, the alternatives are ranked according to this ratio in descending order.

Table 10 presents the rankings of alternatives. Figure 2 depicts the rankings visually. The Spearman Rank Correlation Coefficient method is also applied to obtain the similarities between the alternative rankings found by 9 different methods (Table 11).

**Table 10:** Ranking Results of Nine Different Combinations

alternatives	entropy-PIV	DEMATEL-PIV	CRITIC-PIV	entropy-ARAS	DEMATEL-ARAS	CRITIC-ARAS	entropy-TOPSIS	DEMATEL-TOPSIS	CRITIC-TOPSIS
*i* = 1, Achillea millefolium	4	4	4	4	4	4	4	4	5
i = 2, Plantago major	5	6	5	5	6	5	5	6	4
*i* = 3, Hypericum perforatum	2	2	2	2	2	2	2	2	2
*i* = 4, Malva sylvestris	7	7	7	7	7	7	7	7	7
*i* = 5, Althaea officinalis	9	9	9	9	9	9	9	9	9
*i* = 6, Centella asiatica	1	1	1	1	1	1	1	1	1
*i* = 7, Calendula officinalis	3	3	3	3	3	3	3	3	3
*i* = 8, Momordica charantia	8	8	8	8	8	8	8	8	8
*i* = 9, Tagetes erecta	10	10	10	10	10	10	10	10	10
*i* = 10, Matricaria recutita	6	5	6	6	5	6	6	5	6

**Figure 2:**
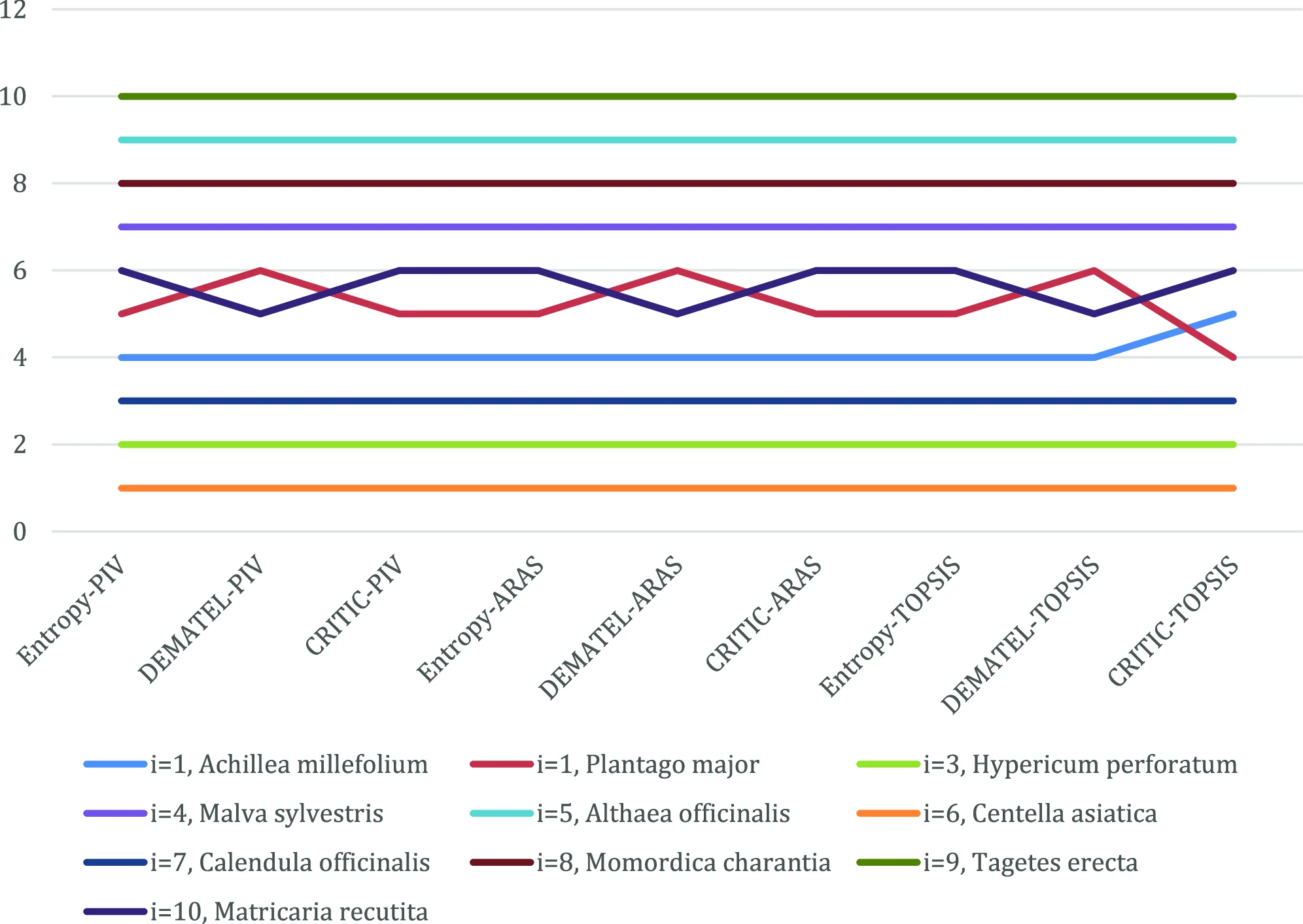
Benchmark of the ranking results.

**Table 11:** Correlation Matrix

	entropy-PIV	DEMATEL-PIV	CRITIC-PIV	Entropy-ARAS	DEMATEL-ARAS	CRITIC-ARAS	Entropy-TOPSIS	DEMATEL-TOPSIS	CRITIC-TOPSIS
entropy-PIV	1.000								
DEMATEL-PIV	0.988	1.000							
CRITIC-PIV	1.000	0.988	1.000						
entropy-ARAS	1.000	0.988	1.000	1.000					
DEMATEL-ARAS	0.988	1.000	0.988	0.988	1.000				
CRITIC-ARAS	1.000	0.988	1.000	1.000	0.988	1.000			
entropy-TOPSIS	1.000	0.988	1.000	1.000	0.988	1.000	1.000		
DEMATEL-TOPSIS	0.988	1.000	0.988	0.988	1.000	0.988	0.988	1.000	
CRITIC-TOPSIS	0.988	0.964	0.988	0.988	0.964	0.988	0.988	0.964	1.000

As observed from Table 10 and Figure 2, nine different combinations of Entropy, CRITIC, DEMATEL weighting methods and PIV, TOPSIS, ARAS alternative ranking methods produce the same three alternatives (*i* = 6, 3, and 7) as the most appropriate ones and exactly the same four alternatives (*i* = 9, 5, 8, and 4) as the least appropriate ones. The three alternatives remaining (*i* = 1, 2, and 10) are ranked in the different positions by different methods but these are not too different. The rank correlations summarized in Table 11 supports these ranking similarities. So, the minimum correlation value computes is 96.4% and most of the rankings have 100% similarity. Therefore, it is concluded that the proposed PiF-DEMATEL-PIV hybrid method is validated because it reveals similar solutions with the other MADM methods under PiF environment. Moreover, it is seen that *C. asiatica* is a robust alternative because it does not lose its highest position in case that different MADM methods are implemented.

## 5 Discussion

Although many minor wounds can heal through the body’s natural repair mechanisms, chronic and nonhealing wounds continue to represent a significant clinical challenge. Factors such as population aging, the growing prevalence of diabetes, vascular complications, and other systemic disorders have contributed to an increasing number of wounds that fail to progress properly through the sequential stages of inflammation, proliferation, and tissue remodeling.^90–92^ Consequently, there is growing interest in developing advanced wound-care materials capable of regulating excessive inflammation and oxidative stress, limiting microbial colonization, and promoting effective tissue regeneration.

Plant-derived extracts and other natural materials are considered promising candidates in this context because they contain a wide range of bioactive compounds that may influence multiple stages of the wound-healing process simultaneously. In addition, they may provide cost-effective and environmentally sustainable alternatives or complementary approaches to conventional wound-care materials.^93–95^ However, identifying the most suitable plant extract remains challenging because different extracts vary considerably in their phytochemical profiles, biological effects, toxicity, availability, and overall healing potential. The PiF-DEMATEL–PIV model applied in this study offers a systematic framework for assessing these competing factors and determining the plant extracts with the greatest overall potential for wound-healing applications. According to the PiF-PIV analysis, *C. asiatica* received the highest ranking among the evaluated plant extracts, followed by *H. perforatum* and *C. officinalis*. The subsequent alternatives were ranked as *A. millefolium*, *M. recutita*, *P. major*, *M. sylvestris*, *M. charantia*, *A. officinalis*, and *T. erecta*. This ranking should be interpreted as an expert-based prioritization derived from the selected wound-healing criteria rather than as evidence from a direct comparative clinical investigation. Nevertheless, the established phytochemical profiles and reported biological activities of the highest-ranked extracts provide a scientific rationale supporting the obtained prioritization.

*C. asiatica* achieved the highest overall performance and contributed particularly strongly to the cell-regeneration and phytochemical-content attributes. This superior performance can be attributed to its ability to promote angiogenesis and regulate key wound repair signals such as collagen synthesis, and further enhance cell renewal.^96^ This finding is consistent with the known phytochemical richness of *C. asiatica*, which contains significant amounts of specific pentacyclic triterpenes, including asiaticoside, madecassoside, asiatic acid, and madecassic acid, as well as polyphenols and flavonoids.^97,98^ Experimental evidence supports the relationship between these bioactive constituents and the high cell-regeneration score obtained in the present analysis. Singkhorn et al. demonstrated that the standardized *C. asiatica*extract ECa 233, with asiaticoside and madecassoside as its major active compounds, enhanced the migration of human HaCaT keratinocytes in a concentration- and time-dependent manner.^99^ This effect was linked to the activation of focal adhesion kinase, Akt, extracellular signal-regulated kinase, and p38 mitogen-activated protein kinase signaling pathways. These molecular pathways are essential for regulating cytoskeletal organization, cell survival, directed cellular migration, and the re-epithelialization process during wound repair. The regenerative activity of madecassoside has also been linked to intracellular Ca^2+^ signaling and the activation of AMPK, mTOR, and ERK-associated pathways.^100^ These findings indicate that the triterpenoid compounds present in *C. asiatica* may contribute beyond general antioxidant effects by actively modulating specific cellular signaling mechanisms involved in migration, proliferation, and tissue regeneration. The phenolic and flavonoid components of *C. asiatica* may further enhance these regenerative effects by contributing to the reduction of oxidative stress. Phenolic compounds can neutralize reactive radical species through hydrogen atom or electron donation, stabilize the resulting phenoxyl radicals via resonance, and chelate transition-metal ions involved in reactive oxygen species generation. Therefore, the strong performance of *C. asiatica* in terms of phytochemical composition, cell regeneration, angiogenic potential, and anti-inflammatory activity may result from the synergistic effects of multiple chemically distinct constituents rather than the action of a single dominant compound.

Among the remaining plant extracts, *H. perforatum* ranked second in terms of overall wound-healing performance, showing its strongest contributions to the phytochemical content and cell regeneration attributes, respectively. Its biological activity is attributed to a diverse range of bioactive compounds, including polyprenylated acylphloroglucinols such as hyperforin, naphthodianthrones such as hypericin and pseudohypericin, biflavonoids such as amentoflavone, flavonol glycosides including rutin and hyperoside, flavonoid aglycones such as quercetin, phenolic acids, tannins, and xanthone derivatives. These constituents contribute through multiple complementary mechanisms. Hyperforin primarily acts through modulation of enzymes and ion channels, whereas flavonoids and phenolic acids contribute more directly to antioxidant activity through radical scavenging, metal chelation, and regulation of redox-associated inflammatory pathways. In addition, hypericin and amentoflavone have been linked to anti-inflammatory effects, while flavonoid and xanthone components may contribute to fibroblast activity, collagen synthesis, and epithelial tissue reconstruction.^101^

The anti-inflammatory properties of hyperforin provide a mechanistic explanation for the strong performance of *H. perforatum* in the present evaluation. Hyperforin has been reported to inhibit 5-lipoxygenase, a key enzyme involved in leukotriene biosynthesis, with an IC_50_ value of approximately 1.6 μM under purified enzyme conditions.^102^ This activity has been associated with interference with the regulatory domain of 5-lipoxygenase and inhibition of its translocation to the nuclear membrane. In addition, hyperforin has been shown to suppress microsomal prostaglandin E_2_ synthase-1 activity at concentrations close to 1 μM, resulting in reduced production of prostaglandin E_2_, an important inflammatory mediatör.^103^ These findings provide a molecular basis for the anti-inflammatory effects associated with hyperforin-containing extracts. Beyond inflammation control, hyperforin may also contribute to re-epithelialization through activation of transient receptor potential canonical 6 (TRPC6) channels. This activation enhances ATP-dependent Ca^2+^ signaling in keratinocytes, promoting cytoskeletal remodeling and directed cell migration toward the wound site.^104^ Therefore, this mechanism establishes a direct connection between a specific *H. perforatum* constituent and the cell-regeneration criterion evaluated in the present model.

The flavonoid and phenolic fractions of *H. perforatum* provide additional antioxidant and tissue-protective effects. Their biological activity is influenced by their molecular structures. Flavonoids containing multiple phenolic hydroxyl groups, catechol-like substitution patterns, and extended conjugated systems generally exhibit greater capacity for radical stabilization. Although glycosylation can enhance polarity and aqueous solubility, it may also influence membrane permeability and the ability of compounds to reach intracellular targets. Similarly, hydroxycinnamic acids, including caffeic and chlorogenic acids, possess conjugated structures that support stabilization of reactive radical intermediates. Experimental studies have demonstrated that *H. perforatum* extracts can enhance fibroblast proliferation, collagen organization, revascularization, and epithelial regeneration.^101,105,106^ Through these effects, *H. perforatum* can accelerate wound healing by promoting epithelial cell migration and supporting collagen synthesis and extracellular matrix remodelling.^107^

*C. officinalis* ranked third and demonstrated strong performance in anti-inflammatory activity, cell regeneration, and phytochemical composition. Its wound-healing potential has been attributed to a diverse range of constituents, including triterpenoids, flavonoids, phenolic acids, carotenoids, polysaccharides, and volatile compounds. Among these, triterpenoids such as faradiol esters, calenduladiol derivatives, lupeol, and oleanolic acid saponins appear to play important roles in its anti-inflammatory and antimicrobial effects.^108^

The flavonoid fraction of *C. officinalis* contains compounds such as quercetin, rutin, narcissin, and isorhamnetin glycosides. These molecules may contribute to oxidative stress regulation and inflammatory control through mechanisms including radical scavenging, transition-metal chelation, and modulation of redox-sensitive signaling pathways. Variations in glycosylation patterns can further influence their polarity, chemical stability, and cellular availability. Carotenoids, including lutein and β-carotene, provide an additional antioxidant mechanism. Their highly conjugated structures enable effective quenching of singlet oxygen and neutralization of lipid-derived radical species, thereby contributing to protection of newly forming tissue from oxidative damage.^83^ The polysaccharide fraction may also support wound repair by enhancing hydration, cellular interactions, and extracellular-matrix organization. Experimental studies have reported that *C. officinalis* extracts can enhance fibroblast activity, epithelial-cell proliferation, granulation-tissue development, collagen arrangement, and extracellular-matrix deposition. They may also regulate matrix metalloproteinase activity, which is essential for controlled degradation and remodeling of damaged tissue.^109–111^

The lower ranking of the remaining extracts should not be interpreted as an indication that they lack wound-healing potential. *A. millefolium*, *M. recutita*, *P. major*, *M. sylvestris*, *M. charantia*, *A. officinalis*, and *T. erecta* contain diverse bioactive compounds, including flavonoids, terpenoids, phenolic acids, iridoid glycosides, mucilaginous components, polysaccharides, and other phytochemicals that may contribute to different phases of the healing process. Their lower overall scores may instead result from less balanced performance across the evaluated criteria, weaker effects in the attributes assigned higher importance within the model, or differences in the quantity and quality of available scientific evidence.

Among the evaluated plant alternatives, phytochemical composition, cell-regeneration potential, and anti-inflammatory activity were the factors most strongly associated with differences in overall performance. Phytochemical content was particularly influential because it provides the chemical foundation underlying many biological effects. Plant-derived triterpenoids, flavonoids, phenolic acids, alkaloids, saponins, tannins, carotenoids, and polysaccharides can participate in multiple mechanisms, including free-radical scavenging, metal-ion chelation, enzyme regulation, membrane interaction, ion-channel modulation, cytokine control, and intracellular signaling regulation.^112,113^

This multifunctional activity is especially relevant for chronic wounds, where microbial burden, oxidative stress, prolonged inflammation, impaired vascularization, and insufficient tissue regeneration frequently coexist. Therefore, chemically diverse plant extracts may influence several components of the wound environment simultaneously. From a translational perspective, the present ranking should also be considered together with the physicochemical behavior of the major active constituents. Differences in polarity, glycosylation, and lipophilicity may affect chemical stability, aqueous solubility, local bioavailability, and release from wound-dressing matrices. Accordingly, hydrogels may be suitable for more polar constituents, whereas liposomes, nanoemulsions, transfersomes, or electrospun nanofibers may improve the dispersion, protection, local retention, and controlled release of less water-soluble or chemically labile compounds. These formulation-related properties were not directly evaluated in the present MADM model and should therefore be confirmed through subsequent experimental studies. Nevertheless, the biological performance of an extract cannot be determined solely by its total phytochemical concentration. The chemical structures of individual constituents, their relative abundance, bioavailability, and potential synergistic or antagonistic interactions are also critical factors influencing the final therapeutic outcome.

The PiF-DEMATEL analysis provided additional understanding of the relative significance of the evaluation criteria. Anti-inflammatory activity was assigned the highest weight (0.150), followed by cell regeneration (0.147) and phytochemical content (0.145). The importance of anti-inflammatory activity and cell regeneration is biologically justified, as effective wound closure requires both the control of excessive or persistent inflammation and the restoration of cellular processes involved in proliferation, migration, collagen synthesis, and re-epithelialization.

Anti-inflammatory activity and cell regeneration were categorized within the “Effect” group, indicating that they represent primarily downstream biological outcomes within the expert-defined interaction network. In contrast, phytochemical content was identified as the most influential criterion within the “Cause” group. Antimicrobial activity, antioxidant activity, and toxicity were also classified as influencing factors, whereas angiogenic activity, anti-inflammatory activity, cell regeneration, and abundance were considered mainly influenced attributes.

This cause–effect organization suggests the presence of a functional hierarchy among the evaluated characteristics. The chemical and biochemical composition of a plant extract forms the foundation for the biological responses observed during wound repair. Phytochemicals may contribute by reducing oxidative stress, modulating inflammatory pathways, limiting microbial growth, regulating cellular signaling, and supporting extracellular-matrix formation. These upstream effects may subsequently influence downstream processes such as cell migration, angiogenesis, inflammatory resolution, and tissue regeneration.

The strongest directional relationship identified by the PiF-DEMATEL analysis was between phytochemical content and cell regeneration, while a substantial influence of phytochemical content on anti-inflammatory activity was also observed. These associations are chemically plausible. Triterpenoids may influence kinase-mediated pathways involved in cell migration and collagen synthesis; flavonoids and phenolic acids may contribute through radical scavenging and suppression of inflammatory mediators; and lipophilic constituents may interact with cellular membranes, ion channels, or intracellular signaling proteins.

However, these relationships should not be interpreted as evidence that wound regeneration is determined directly by the total amount of phytochemicals present. The DEMATEL outcomes represent structured expert assessments of the relationships among selected criteria. Moreover, total phytochemical concentration does not differentiate between highly bioactive compounds and constituents with limited biological relevance. An extract containing a lower overall phytochemical quantity may still demonstrate strong biological activity if it possesses potent individual compounds or favorable combinations of chemically complementary constituents.

Toxicity showed the lowest degree of variation among the evaluated alternatives. This observation does not indicate that toxicity is insignificant or that all extracts possess equivalent safety profiles. Rather, it reflects the relatively similar toxicity evaluations assigned by the experts to the selected alternatives. Since plant-derived compounds often exhibit concentration-dependent effects, certain constituents may become cytotoxic, sensitizing, or irritating at elevated concentrations. Therefore, toxicity evaluation remains essential during subsequent experimental validation.

Overall, the findings demonstrate a chemically consistent relationship between plant-extract composition and predicted wound-healing performance. Anti-inflammatory activity and cell regeneration were identified as the most important biological outcomes, whereas phytochemical content emerged as the primary upstream factor influencing these responses. The high rankings of *C. asiatica*, *H. perforatum*, and *C. officinalis* are consistent with the complementary activities reported for their triterpenoids, acylphloroglucinols, flavonoids, phenolic acids, carotenoids, and related bioactive constituents.

Nevertheless, these results should be interpreted as a prioritization framework rather than direct experimental proof of superiority. The PiF-DEMATEL–PIV model provides a systematic approach for identifying promising candidates and reducing the number of extracts requiring further detailed laboratory investigation.

## 6 Conclusions

The present study has successfully utilized a new MADM framework within a Picture fuzzy set extended decision-making environment to evaluate the efficacy of the various plant extracts as eco-friendly, cost-effective and innovation wound healing materials. While global burden of chronical wounds increases due to aging population and diabetic complications, the search for functional biomaterials has never been that critical.

First, the analysis defines *C. asiatica* as the most effective wound healing alternative due to its outstanding contribution to cell regeneration and angiogenic activity. These results have been supported by high levels of triterpenes and flavonoids which managing wound repair signals of CA like collagen. *H. perforatum* and *C. officinalis* ranked as the second and third most efficient alternatives, respectively, performing very close to the best. While *Hypericum* outperforms in accelerating the healing process via rapid collagen restructuring, Calendula performs as a powerful anti-inflammatory multipurpose agent by quietening the inflammation signals effectively in order to facilitate faster wound closure.

Phytochemical content, antimicrobial activity, and antioxidant activity have been determined as mainly effectual attributes for wound healing. These attributes directly dominate the success of affected results such as cell regeneration and anti-inflammatory effects. Phytochemical content aroused as the most critical attribute in all species, and it almost showed a linear relation with the increase in cell generation rates. Of all plant extracts assessed, toxicity have been found to be as the least affected attribute, demonstrating that these alternatives obtained from nature exhibit a wide safety profile.

However, even with such a strong theoretical framework, the study suffers from some limitations which should be considered. First, even though the Picture Fuzzy environment is successful in dealing with uncertainty and hesitation, the data used is based on subjective expert opinions and heuristics derived from the literature, not actual experimental measurements at the time of the study. Second, the phytochemical composition and the actual strength of natural plant extracts depend largely on the location of their origin, season of harvest, and method of extraction, which might affect their clinical efficiency.

Further research should concentrate on validating the mathematical results of the decision-making process empirically. Among the suggestions would be creation of novel biopolymeric drug carriers like electrospun nanofibers or polysaccharide hydrogels carrying the selected *C. asiatica* extract to perform controlled in vitro and in vivo wound healing assays. Besides, investigation into the possible synergy between the selected three candidates for wound healing treatment, including *C. asiatica*, *H. perforatum*, and *Chelidonium officinalis*, would lead to potent wound healing drugs targeting multiple targets at once.

## Appendix A

### Expert judgments for DEMATEL

*e* = 1	*j* = 1	*j* = 2	*j* = 3	*j* = 4	*j* = 5	*j* = 6	*j* = 7	*j* = 8
*j* = 1	1	0.7	0.6	NO	NO	NO	NO	NO
*j* = 2	0.8	1	0.4	NO	NO	NO	0.2	NO
*j* = 3	0.7	0.5	1	NO	0.3	0.4	NO	0.2
*j* = 4	0.7	0.5	0.7	1	0.8	0.9	0.7	0.3
*j* = 5	0.7	0.5	0.5	0.7	1	0.5	0.4	0.2
*j* = 6	0.5	0.5	0.6	0.9	0.4	1	0.5	0.3
*j* = 7	NO	NO	NO	NO	NO	NO	1	NO
*j* = 8	0.7	0.5	0.5	0.3	0.5	0.4	NO	1
*e* = 2	*j* = 1	*j* = 2	*j* = 3	*j* = 4	*j* = 5	*j* = 6	*j* = 7	*j* = 8
*j* = 1	1	0.8	0.8	0.8	0.6	0.9	NO	0.6
*j* = 2	0.8	1	0.7	0.7	No	0.4	NO	0.6
*j* = 3	0.8	0.7	1	0.7	0.6	0.8	NO	0.4
*j* = 4	0.75	0.6	0.7	1	0.7	0.8	NO	0.6
*j* = 5	0.6	No	0.6	0.7	1	0.7	NO	0.3
*j* = 6	0.9	0.7	0.8	0.8	0.7	1	NO	0.4
*j* = 7	NO	NO	NO	NO	NO	NO	1	NO
*j* = 8	0.6	0.6	0.4	0.6	0.3	0.4	NO	1
*e* = 3	*j* = 1	*j* = 2	*j* = 3	*j* = 4	*j* = 5	*j* = 6	*j* = 7	*j* = 8
*j* = 1	1	0.75	0.7	NO	ABS	ABS	NO	0.2
*j* = 2	0.75	1	0.5	NO	ABS	ABS	NO	0.1
*j* = 3	0.8	0.5	1	NO	0.4	0.5	NO	0.2
*j* = 4	0.8	0.4	0.75	1	0.8	0.8	NO	NO
*j* = 5	0.6	0.3	0.5	0.8	1	0.6	NO	0.2
*j* = 6	0.6	0.45	0.5	0.8	0.5	1	NO	0.4
*j* = 7	NO	NO	NO	NO	NO	NO	1	NO
*j* = 8	0.7	0.5	0.55	0.2	0.6	0.4	NO	1
*e* = 4	*j* = 1	*j* = 2	*j* = 3	*j* = 4	*j* = 5	*j* = 6	*j* = 7	*j* = 8
*j* = 1	1	0.7	0.6	0.7	0.7	0.7	NO	NO
*j* = 2	0.85	1	0.5	0.8	NO	NO	NO	NO
*j* = 3	0.8	0.6	1	0.7	0.7	0.6	NO	NO
*j* = 4	0.7	0.5	0.8	1	0.8	0.9	NO	0.4
*j* = 5	0.7	0.5	0.6	0.8	1	NO	NO	0.3
*j* = 6	0.6	0.5	0.8	0.8	NO	1	NO	ABS
*j* = 7	NO	NO	NO	NO	NO	NO	1	NO
*j* = 8	0.7	0.6	0.6	0.6	0.3	0.2	NO	1
*e* = 5	*j* = 1	*j* = 2	*j* = 3	*j* = 4	*j* = 5	*j* = 6	*j* = 7	*j* = 8
*j* = 1	1	0.85	0.7	0.75	0.6	0.9	No	0.5
*j* = 2	0.85	1	0.65	0.5	0.45	0.7	No	0.2
*j* = 3	0.7	0.65	1	0.75	0.4	0.65	No	0.5
*j* = 4	0.75	0.5	0.75	1	0.6	0.8	No	0.3
*j* = 5	0.6	0.45	0.4	0.6	1	0.4	No	0.5
*j* = 6	0.9	0.7	0.65	0.8	0.4	1	No	0.2
*j* = 7	No	No	No	No	No	No	1	No
*j* = 8	0.5	0.2	0.5	0.3	0.5	0.2	No	1

## Appendix B

### Expert judgments for PIV

*e* = 1	*j* = 1	*j* = 2	*j* = 3	*j* = 4	*j* = 5	*j* = 6	*j* = 7	*j* = 8
*i* = 1	0.7	0.5	0.8	0.8	0.7	0.8	0.9	0.1
*i* = 2	0.7	0.4	0.8	0.8	0.6	0.75	0.95	0.03
*i* = 3	0.9	0.7	0.9	0.9	0.8	0.8	0.8	0.2
*i* = 4	0.6	0.4	0.7	0.7	0.5	0.8	0.8	0.05
*i* = 5	0.7	0.4	0.7	0.7	0.4	0.7	0.8	0.05
*i* = 6	0.9	0.8	0.9	0.9	0.7	0.8	0.7	0.1
*i* = 7	0.85	0.7	0.8	0.8	0.7	0.8	0.8	0.07
*i* = 8	0.8	0.6	0.8	0.7	0.6	0.8	0.8	0.2
*i* = 9	0.6	0.5	0.6	0.7	0.7	0.75	0.6	0.1
*i* = 10	0.7	0.5	0.9	0.8	0.6	0.8	0.8	0.08
*e* = 2	j = 1	j = 2	j = 3	j = 4	j = 5	j = 6	j = 7	j = 8
*i* = 1	0.7	0.6	0.8	0.8	0.7	0.75	0.6	0.2
*i* = 2	0.7	0.5	0.8	0.7	0.65	0.7	0.8	0.1
*i* = 3	0.7	0.6	0.65	0.85	0.7	0.75	0.6	0.4
*i* = 4	0.6	0.5	0.75	0.7	0.6	0.7	0.6	0.1
*i* = 5	0.65	0.45	0.7	0.6	0.4	0.55	0.6	0.05
*i* = 6	0.95	0.9	0.85	0.9	0.6	0.85	0.7	0.2
*i* = 7	0.85	0.8	0.85	0.8	0.7	0.8	0.7	0.15
*i* = 8	0.6	0.55	0.6	0.6	0.6	0.7	0.5	0.3
*i* = 9	0.4	0.35	0.5	0.55	0.6	0.5	0.6	0.2
*i* = 10	0.75	0.6	0.85	0.8	0.65	0.8	0.7	0.2
*e* = 3	j = 1	j = 2	j = 3	j = 4	j = 5	j = 6	j = 7	j = 8
*i* = 1	0.65	0.65	0.8	0.85	0.7	0.8	0.8	0.08
*i* = 2	0.75	0.5	0.8	0.85	0.65	0.8	0.9	0.1
*i* = 3	0.95	0.75	0.85	0.9	0.8	0.85	0.7	0.3
*i* = 4	0.7	0.55	0.8	0.8	0.55	0.75	0.8	0.05
*i* = 5	0.8	0.5	0.8	0.85	0.5	0.65	0.7	0.05
*i* = 6	0.98	0.8	0.75	0.9	0.65	0.8	0.7	0.15
*i* = 7	0.8	0.7	0.8	0.7	0.7	0.7	0.8	0.1
*i* = 8	0.85	0.65	0.75	0.8	0.6	0.7	0.6	0.3
*i* = 9	0.6	0.6	0.6	0.7	0.65	0.65	0.6	0.1
*i* = 10	0.65	0.55	0.8	0.75	0.6	0.75	0.7	0.15
*e* = 4	j = 1	j = 2	j = 3	j = 4	j = 5	j = 6	j = 7	j = 8
*i* = 1	0.8	0.6	0.8	0.9	0.6	0.85	0.9	0.2
*i* = 2	0.8	0.6	0.8	0.9	0.6	0.8	0.9	0.1
*i* = 3	0.8	0.7	0.8	0.9	0.8	0.8	0.8	0.3
*i* = 4	0.8	0.6	0.8	0.8	0.5	0.8	0.8	0.1
*i* = 5	0.7	0.5	0.7	0.8	0.5	0.75	0.8	0.1
*i* = 6	0.9	0.85	0.8	0.9	0.6	0.75	0.7	0.1
*i* = 7	0.8	0.7	0.8	0.7	0.6	0.7	0.9	0.15
*i* = 8	0.7	0.65	0.6	0.7	0.6	0.7	0.8	0.3
*i* = 9	0.7	0.6	0.6	0.6	0.6	0.6	0.7	0.2
*i* = 10	0.8	0.6	0.8	0.8	0.6	0.8	0.8	0.15
*e* = 5	j = 1	j = 2	j = 3	j = 4	j = 5	j = 6	j = 7	j = 8
*i* = 1	0.7	0.4	0.8	1	0.6	0.8	0.8	0.2
*i* = 2	0.6	0.3	0.7	1	0.6	0.7	0.8	0.1
*i* = 3	0.8	0.7	0.8	1	0.7	0.8	1	0.5
*i* = 4	0.6	0.4	0.8	1	0.6	0.8	1	0.2
*i* = 5	0.5	0.4	0.7	0.9	0.4	0.5	0.7	0.1
*i* = 6	0.85	0.8	0.8	1	0.5	0.7	0.5	0.2
*i* = 7	0.7	0.6	0.8	1	0.6	0.8	0.7	0.2
*i* = 8	0.5	0.4	0.7	1	0.6	0.7	0.8	0.4
*i* = 9	0.6	0.5	0.7	1	0.6	0.8	0.8	0.2
*i* = 10	0.6	0.7	0.8	1	0.6	0.7	0.5	0.2
